# Metabolic inflammation at the adipose-brain axis

**DOI:** 10.3389/fphys.2026.1795058

**Published:** 2026-04-10

**Authors:** Saudina Mateus-Gomes, Amnah Al-Sayyar, Baptiste Lobey, Agnes Nadjar, Rejane Rua, Rasheed Ahmad

**Affiliations:** 1 Centre d’Immunologie de Marseille-Luminy, Centre national de la recherche scientifique (CNRS) Institut national de la santé et de la recherche médicale (INSERM), Aix Marseille Université, Marseille, France; 2 Immunology & Microbiology Department, Dasman Diabetes Institute, Kuwait, Kuwait; 3 Institute of Neurodegenerative Diseases, Centre national de la recherche scientifique (CNRS) UMR-5293, University of Bordeaux, Bordeaux, France; 4 Institut Universitaire de France (IUF), Paris, France

**Keywords:** adipocyte, adipokines, brain, neuroinflammation, obesity

## Abstract

Overweight and obesity have emerged as global health crises and are increasingly recognized as drivers of central nervous system (CNS) dysfunction. Beyond excess energy storage, white adipose tissue (WAT) functions as an active endocrine and immune organ that, during obesity, undergoes inflammatory remodeling and releases cytokines, lipid mediators, adipokines, and extracellular vesicles that influence brain physiology. These peripheral signals disrupt key brain interfaces, including the blood-brain barrier (BBB), perivascular and glymphatic clearance pathways, promoting endothelial dysfunction, altered astrocyte-pericyte support, impaired amyloid-β clearance, and region-specific glial activation. Obesity-associated neuroinflammation is characterized by microglial priming and astrocyte reactivity across the hypothalamus, hippocampus, and other circuits governing metabolism, cognition, and reward, with growing evidence for sex-dependent vulnerability. We further highlight adipokines as key mediators of adipose-brain communication. In obesity, leptin resistance impairs central energy regulation, reduced adiponectin contributes to neuroinflammation and synaptic dysfunction, and elevated resistin enhances TLR4-dependent inflammatory signaling and BBB permeability, collectively linking metabolic stress to neurodegenerative processes. Finally, we review therapeutic strategies targeting the adipose-brain axis, including exercise and dietary interventions that improve neuroplasticity and barrier integrity, and pharmacological approaches such as orlistat and incretin-based therapies. Emerging multi-incretin agonists, including tirzepatide and retatrutide, raise important questions regarding direct CNS actions beyond metabolic benefits, underscoring the need to integrate barrier biology and neuroimmune mechanisms in future studies.

## 1 Introduction

**Figure 1:**
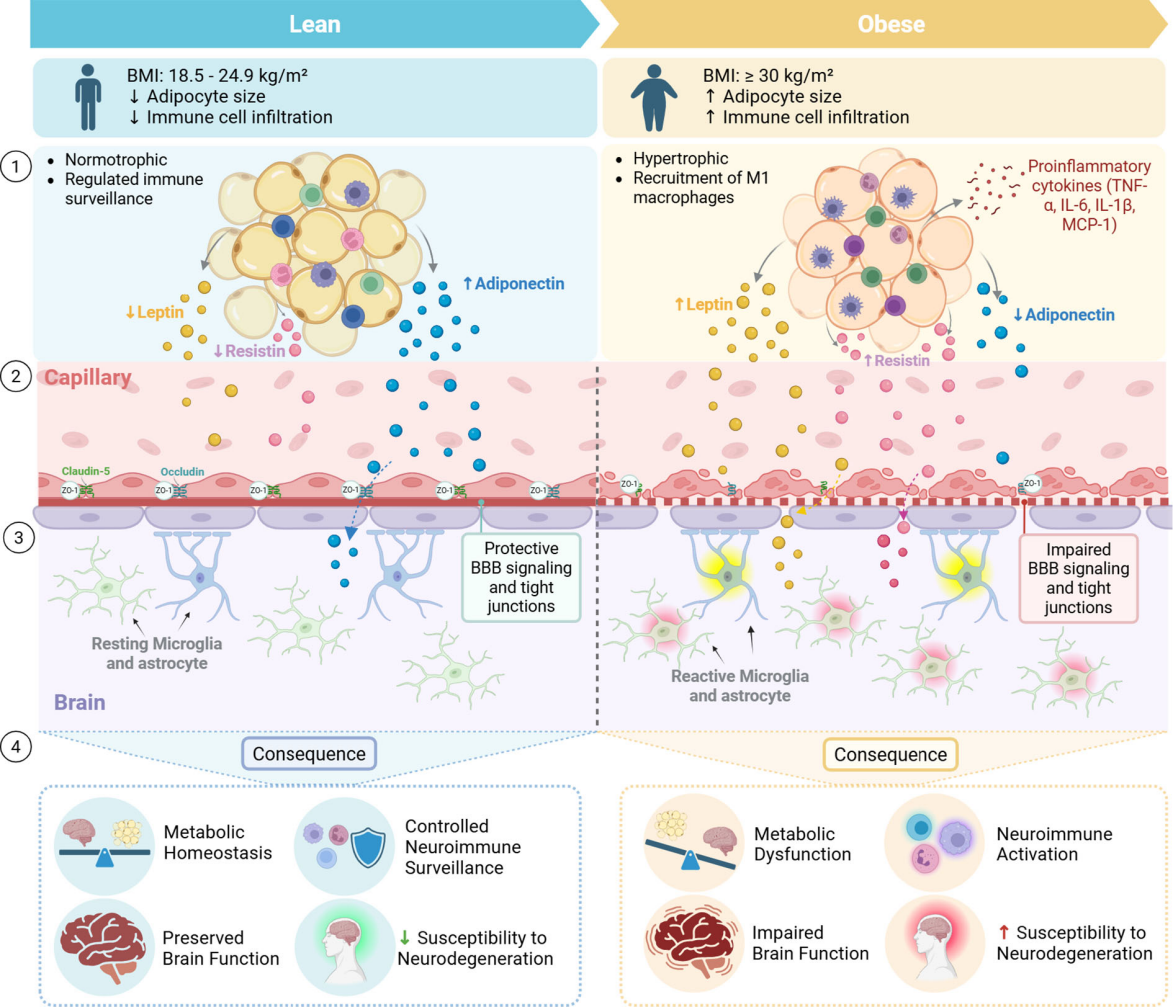
Adipose–brain communication in lean and obese states. Schematic representation of adipose tissue–brain crosstalk organized into four levels (1–4). (1) In lean conditions, white adipose tissue (WAT) is composed of normotrophic adipocytes with regulated immune surveillance and a favorable adipokine profile (physiological leptin, low resistin, high adiponectin), whereas obesity is characterized by adipocyte hypertrophy, M1 macrophage recruitment, and increased secretion of pro-inflammatory cytokines. (2) These adipose-derived signals enter the circulation and reach the brain vasculature. (3) In lean states, adipokines support protective BBB signaling with preserved tight-junction integrity (claudin-5, occludin, ZO-1) and resting glial cells, while obesity induces impaired BBB signaling, tight-junction disruption, and reactive microglia and astrocytes.(4) Functionally, lean adipose–brain communication maintains metabolic homeostasis, controlled neuroimmune surveillance, and preserved brain function, with reduced susceptibility to neurodegeneration, whereas obesity promotes metabolic dysfunction, neuroimmune activation, impaired brain function, and increased neurodegenerative vulnerability. Created in https://BioRender.com.

Overweight and obesity are recognized as a global pandemic and are associated with increased morbidity, mortality, and reduced quality of life (De et al., 2023). These conditions are defined by excessive accumulation of body fat, corresponding to a body mass index (BMI) of ≥30 kg/m² (Avgerinos et al., 2019). According to the World Health Organization (WHO), adult obesity has doubled while adolescent obesity has quadrupled since 1990 (World Health Organization, 2025). Projections further estimate that by 2030, nearly 17% of men and 22% of women in the world population will be living with obesity (World Obesity Federation, 2025). A central driver of the pathological consequences of obesity is adipose tissue, which plays a key endocrine and immunological role (Salas-Venegas et al., 2022). Adipose tissue exists in two main forms: brown adipose tissue (BAT) and white adipose tissue (WAT). BAT is abundant in newborns but decreases with age, while WAT is the predominant adipose depot in adults and is primarily responsible for the metabolic and inflammatory complications linked to obesity (Salas-Venegas et al., 2022). During obesity, the excessive expansion of WAT increases the risk of multiple chronic diseases, including osteoarthritis, diabetes, hypertension, and asthma; as its endocrine activity becomes dysregulated (De et al., 2023). This inflammatory state promotes endothelial dysfunction, blood-brain barrier (BBB) disruption, and oxidative stress, ultimately leading to neurodegenerative diseases, with emerging evidence suggesting sex-specific differences in vulnerability and outcomes (**Figure 1**) (Salas-Venegas et al., 2022). Hypertrophic adipocytes promote immune cell recruitment and inflammatory remodeling of adipose tissue (Salas-Venegas et al., 2022). This is validated clinically as obese individuals exhibited hypoxia, hypertrophy, and increased infiltration of inflammatory macrophages compared to healthy individuals in their subcutaneous WAT (Fisk et al., 2022). However, these circulating inflammatory mediators not only influence adipose-brain communication but are also shaped by brain derived neuroendocrine and autonomic signals, highlighting a bidirectional adipose-brain axis (Huang et al., 2022; Till et al., 2022).

In the central nervous system (CNS), obesity is recognized as a major risk factor for neuroinflammatory responses across multiple brain regions, including the hypothalamus, hippocampus, cerebellum, amygdala, and cerebral cortex (Salas-Venegas et al., 2022). Evidence indicates that obesity associated inflammatory signals can activate microglia and astrocytes, increase the release of neurotoxic mediators, and alter glial metabolism, collectively contributing to neuroinflammation (Lee et al., 2018). One of the first brain regions affected by peripheral metabolic and inflammatory signals is the hypothalamus. Studies on mice indicate that lipid metabolites such as long-chain saturated fatty acids promote adipose tissue expansion, cross the BBB, and accumulate in the hypothalamus, where they are sensed by resident microglia (Valdearcos et al., 2014; Jais and Brüning, 2017). This lipid sensing engages innate immune pathways, including TLR4-MyD88 dependent activation of NF-κB signaling, leading to microglial activation, pro-inflammatory cytokine production, and gliosis, which in turn disrupt hypothalamic neuronal circuits, impair energy homeostasis and dysregulate insulin and leptin signaling (Jais and Brüning, 2017; Valdearcos et al., 2017).

Adipose tissue is a highly heterogeneous organ composed not only of adipocytes but also a diverse stromal and immune cell compartment, including macrophages, T cells, B cells, and innate lymphoid cells (Corvera, 2021). In the context of obesity, this immune landscape is remodeled, with expansion and phenotypic reprogramming of adipose tissue macrophages (ATMs) and other immune populations that drive chronic low-grade inflammation. Chronic exposure to high-fat diet (HFD) promotes perivascular and parenchymal macrophage proliferation and expansion in the hypothalamus resulting in a state of chronic hypothalamic inflammation marked by elevated iNOS expression, increased pro-inflammatory cytokine levels, and disruption of BBB integrity in mice (Lee et al., 2018). Beyond the hypothalamus, obese individuals exhibit reduced dopaminergic and serotonergic modulation in the midbrain and pons, respectively, affecting the regulation of food intake. This is reflected by altered dopaminergic and serotonergic innervation of the hypothalamus, thalamus, and amygdala, thereby influencing feeding behavior (Tomasi et al., 2009). Together, these findings highlight obesity as a condition that extends far beyond metabolic dysregulation, exerting direct and indirect effects on the CNS through adipose derived inflammatory signaling. In addition to the peripheral-central view, recent reviews have proposed an alternative framework in which obesity is initiated by primary dysfunction of brain circuits controlling energy balance, particularly through nutrient and inflammation driven microglial alterations in the hypothalamus, with adipose tissue expansion and inflammation emerging as downstream consequence (AL-Dalaeen and AL-Domi, 2022; Cutugno et al., 2024). However, the concept of obesity as a brain disease remains an area of active debate, and the relative contribution of central versus peripheral drivers of obesity likely depends on disease stage, environmental context, and individual susceptibility. Importantly, unlike the progressive and immune cell infiltrative neuroinflammation observed in demyelinating or advanced neurodegenerative diseases, the low-garde inflammatory state of obesity mainly reflects microglial priming, astrocyte reactivity, altered cytokine production, and metabolic signaling changes rather than overt demyelination or widespread neuronal loss (Guillemot-Legris and Muccioli, 2017; Rohm et al., 2022; Ramírez-Carreto et al., 2023). Both preclinical HFD models and human neuroimaging studies support the presence of region-specific glial activation and subtle BBB alterations in the absence of massive leukocyte infiltration (Thaler et al., 2012; André et al., 2017; Valdearcos et al., 2017; Sewaybricker et al., 2023; Feng et al., 2024; Plantera et al., 2025). Thus, obesity-driven neuroinflammation represents a glial remodeling process distinct from the antigen-driven and tissue-destructive inflammation observed in autoimmune disorders such as multiple sclerosis, and from the aggregate-driven neuroinflammation in advanced neurodegenerative disease (Zhang et al., 2023). This review explores how adipose tissue-derived metabolic and inflammatory cues interact with the brain to influence CNS function and drive the initiation and progression of obesity-associated neuroinflammation.

## 2 Adipose tissue and the blood-brain barrier

At the interface between the periphery and the CNS lies one of the most critical protective structures, the BBB. It is formed by endothelial cells connected through tight junctions, supported by pericytes, astrocytic end-feet, and an organized extracellular matrix within the neurovascular unit (Salas-Venegas et al., 2022). This complex structure plays an essential gatekeeping role, regulating the exchange of molecules between the circulation and the brain along with maintaining CNS homeostasis (Salas-Venegas et al., 2022). By controlling nutrient transport, restricting entry of neurotoxins, and preserving ionic balance, the BBB ensures a stable microenvironment for optimal brain function (Salas-Venegas et al., 2022). In obesity, prolonged HFD exposure induces BBB leakage, largely through inflammatory mechanisms that disrupt endothelial integrity (Feng et al., 2024). This includes elevated pro-inflammatory cytokines and oxidative stress which disrupts the expression and organization of tight junction proteins such as claudin-5, occludin, and ZO-1, ultimately increasing endothelial permeability (**Figure 1**) (Feng et al., 2024). Therefore, the BBB becomes more permeable, allowing neurotoxic molecules and peripheral immune cells to infiltrate the brain parenchyma, leading to neuroinflammation (Feng et al., 2024). Moreover, pericytes are similarly affected by obesity, as these mural cells play a role in maintaining vascular stability and regulating BBB function (Rustenhoven et al., 2017). Obesity induced metabolic and inflammatory stress disrupts pericyte function, promoting oxidative damage and pericyte apoptosis (Rhea et al., 2017). This impairs survival pathways such as PDGF/PDGFR and Notch signaling reducing pericyte-endothelial interactions and compromising vascular support (Rhea et al., 2017). The resulting loss of pericyte coverage weakens tight junction integrity, increases BBB permeability, and facilitates the entry of neurotoxic factors into the CNS. Pericyte dysfunction also disrupts cerebral blood flow regulation and induces neuroinflammation, increasing vulnerability to neurodegenerative disease (Rhea et al., 2017; Salas-Venegas et al., 2022; Feng et al., 2024).

## 3 Adipose tissue and the glymphatic system

**Figure 2:**
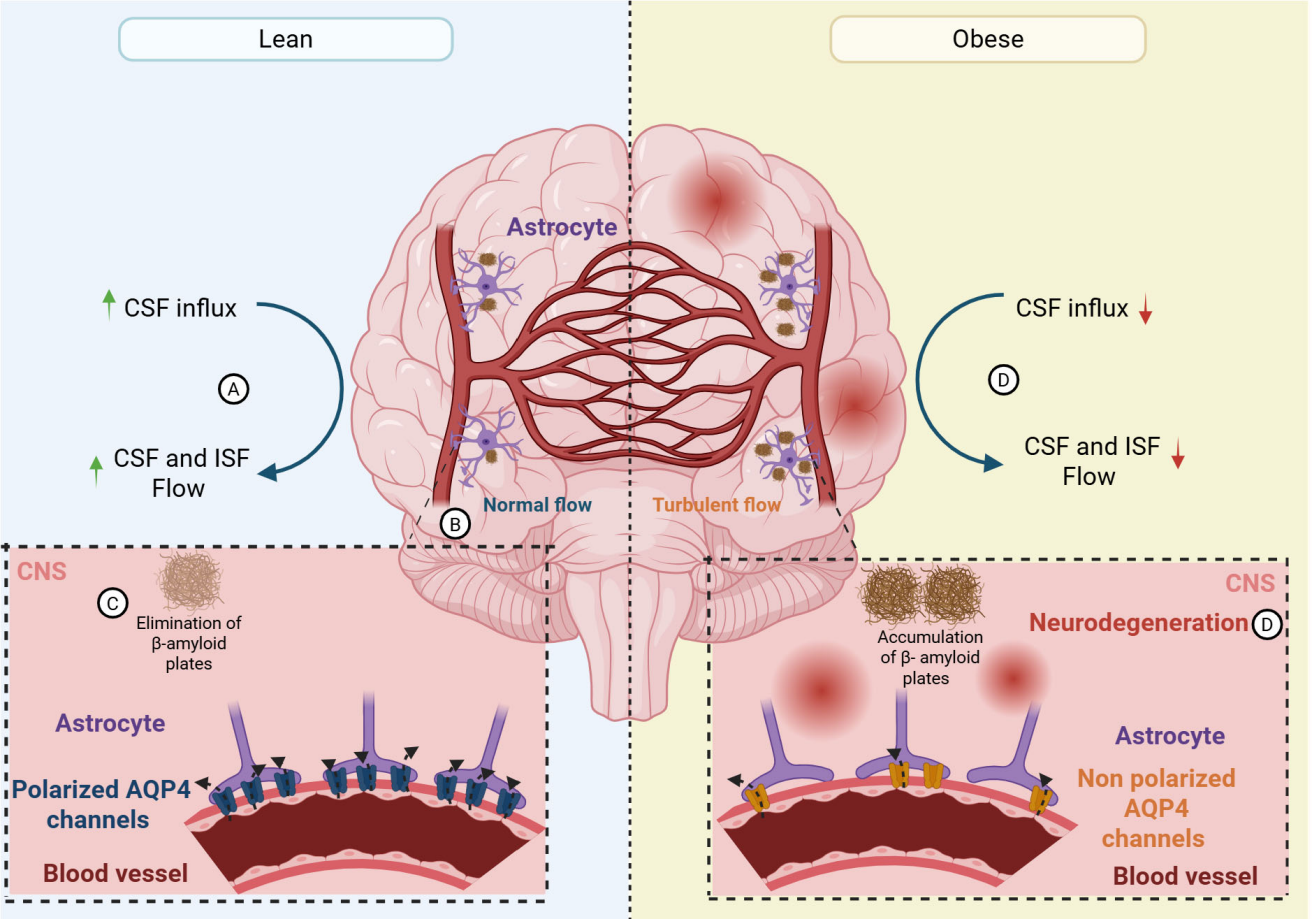
Obesity associated disruption of glymphatic function impairs brain waste clearance. Schematic representation comparing glymphatic activity in lean (left) and obese (right) conditions. In the lean brain, efficient cerebrospinal fluid (CSF) influx and coordinated CSF–interstitial fluid (ISF) exchange promotes normal glymphatic flow **(A, B)**, facilitating effective clearance of metabolic waste, including β-amyloid peptides **(C)**. This process is supported by polarized astrocytes with perivascular localization of aquaporin-4 (AQP4) channels at astrocytic end-feet. In contrast, obesity is associated with reduced CSF influx and impaired CSF–ISF exchange **(D)**, resulting in disrupted glymphatic flow and diminished waste clearance. Loss of astrocyte polarity and disruption of AQP4 channels contribute to β-amyloid accumulation and promote neurodegenerative processes within the CNS. Created in https://BioRender.com.

Another critical component of CNS clearance is the glymphatic system, first described in 2012. This system comprises a specialized perivascular space (PVS) that enables rapid exchange between cerebrospinal fluid (CSF) and interstitial fluid (ISF) (Sun et al., 2025). It plays an essential role in facilitating the removal of metabolic waste and distributing nutrients throughout the brain (Sun et al., 2025). Under physiological conditions, the glymphatic system drains over 60% of brain amyloid-β (Aβ) to the lymphatic system through convective flow driven by arterial pulsations (Morris et al., 2014). Obesity is associated with reduced glymphatic activity, impaired CSF-ISF exchange, and diminished clearance of neurotoxic metabolites such as Aβ (**Figure 2**) (André et al., 2017; Zhang et al., 2023). Since obesity-induced neuroinflammation increases the risk of neurodegenerative disorders, obese individuals with Parkinson’s disease (PD) exhibit greater glymphatic dysfunction, as assessed by diffusion tensor image analysis along the PVS (DTI-ALPS), compared to lean individuals, with the degree of impairment correlating with motor symptom severity (Rhea et al., 2017). Furthermore, growing evidence indicates that metabolic dysfunction is a consequence of obesity. Studies demonstrated that metabolic disorders such as diabetes impair glymphatic function, contributing to reduced clearance of metabolic waste and cognitive decline (Wardlaw et al., 2013). Clinical studies further reveal an association between BMI and glymphatic efficiency in patients with neurodegenerative diseases (Tian et al., 2024), suggesting that excess adiposity itself negatively impacts glymphatic flow. Consistently, patients with Alzheimer’s disease (AD) and type 2 diabetes mellitus (T2DM) exhibit enlarged PVS which is an indication of glymphatic impairment, compared to patients with AD alone (Sun et al., 2025).

Complementing these clinical observations, experimental studies identify glymphatic dysfunction as a central mechanistic link between metabolic disease and neurodegeneration. A HFD-induced model shows reduced CSF influx, impaired clearance of neurotoxic proteins such as Aβ, and disrupted astrocytic Aquaporin-4 (AQP4) polarization, changes that coincide with BBB breakdown, neuroinflammation, and cognitive deficits (Zhan et al., 2024). Moreover, inducing long-term HFD in mice did not globally impair glymphatic flow; instead, glymphatic influx was preserved across most brain regions and was specifically increased in the hypothalamus (Delle et al., 2023). In this model, the hypothalamus showed enhanced CSF tracer influx along with elevated AQP4 vascular polarization, suggesting a region-specific upregulation of glymphatic clearance in response to chronic metabolic stress (Delle et al., 2023). These changes occurred alongside neuroinflammation and behavioral alterations in obese mice, indicating that metabolic alteration of hypothalamic neurons and glia may dynamically modulate local waste clearance pathways in obesity rather than uniformly suppress glymphatic function (Delle et al., 2023). Furthermore, pharmacological restoration of glymphatic function using ethanol extracts of Alisma orientale (EEAO) in obese mice improved Aβ clearance and cognitive deficits, identifying glymphatic dysfunction as a key mediator of obesity-associated cognitive impairment (Zhan et al., 2024). Moreover, in db/db mice (i.e. severe obesity and T2DM), excessive activation of MMP-9/β-dystroglycan disrupted astrocytic AQP4 anchoring, leading to increased endocytosis-lysosomal degradation of AQP4 and associated cognitive impairment, whereas inhibition of this pathway restored its levels and partially alleviated cognitive deficits, highlighting its therapeutic potential in diabetes-related cognitive dysfunction (Yuan et al., 2024). Furthermore, obese Zucker rats (i.e. a genetic model of metabolic syndrome) exhibited BBB abnormalities marked by increased AQP4 and altered GLUT1 regulation, changes that are highly relevant to glymphatic function and may indirectly indicate glymphatic dysregulation (Tomassoni et al., 2020). Collectively, obesity driven metabolic and inflammatory stress emerges as a critical determinant of glymphatic dysfunction, reshaping perivascular clearance mechanisms in ways that favor neurodegeneration.

## 4 Metabolic inflammation as a driver of brain dysfunction

Obesity-associated metabolic inflammation represents a systemic immune state driven by adipose tissue remodeling and metabolic stress. This condition is characterized by sustained cytokine production, inflammasome activation, and dysregulated lipid mediator synthesis, which collectively reshape peripheral immune signaling and exert downstream effects on endothelial integrity, BBB function, and glial activation within the CNS.

### 4.1 Cytokines

At the tissue level, this inflammatory state originates within expanding adipose depots, where adipocyte hypertrophy induces local hypoxia, lipotoxicity, and adipocyte death (Kawai et al., 2021). These alterations promote recruitment and activation of ATMs, which progressively shift toward a pro-inflammatory phenotype and accumulate around necrotic adipocytes, forming crown-like structures (Kawai et al., 2021). Both hypertrophic adipocytes and ATMs contribute to the production of key pro-inflammatory cytokines, including TNF-α, IL-6, and IL-1β, through interconnected molecular pathways (Huang et al., 2012; Kawai et al., 2021). TNF-α is primarily produced by macrophages in response to excess free fatty acids (FFAs) and danger-associated molecular patterns (DAMPs) released by stressed adipocytes (Huang et al., 2012). Saturated fatty acids activate Toll-like receptor 4 (TLR4), triggering MyD88-dependent signaling and downstream NF-κB and JNK activation, thereby promoting TNF-α transcription in macrophages (Tomassoni et al., 2020). In diet-induced obesity (DIO) models, HFD feeding enhances this signaling cascade within ATMs, reinforcing TLR4-driven inflammatory amplification (Tomassoni et al., 2020). Furthermore, IL-6 is produced by both adipocytes and macrophages in response to metabolic stress and inflammatory stimuli, including TNF-α signaling (Kawai et al., 2021). Single-cell RNA sequencing analyses of WAT from obese individuals and HFD-fed mice demonstrate IL-6 transcript expression across adipocyte populations and immune cell subsets (Kawai et al., 2021). Consistently, isolated adipocytes from obese individuals exhibited increased IL-6 secretion compared to lean controls, confirming enhanced functional cytokine output (Kawai et al., 2021). In addition, TNF-α, activation of NF-κB in adipocytes has been shown to drive IL-6 expression in response to pro-inflammatory stimuli *in vitro*, where palmitate activates NF-κB signaling and enhances IL-6 expression (Ajuwon and Spurlock, 2005). In contrast, IL-1β secretion is tightly regulated and largely restricted to immune cells such as macrophages, requiring activation of the NLRP3 inflammasome that is usually triggered by lipid overload, mitochondrial dysfunction, and reactive oxygen species (ROS) production in obesity (Ajuwon and Spurlock, 2005). This involves formation of the NLRP3 complex, recruitment of the adaptor protein ASC, and activation of caspase-1, which cleaves pro-IL-1β into its mature, bioactive form (Ajuwon and Spurlock, 2005). In HFD mouse models, genetic deletion of NLRP3 or caspase-1 reduces IL-1β levels and improves metabolic parameters, supporting a causal role for inflammasome activation in obesity-associated inflammation (Ajuwon and Spurlock, 2005). In the context of the CNS, chronic HFD feeding increases central pro−inflammatory cytokine levels in brain regions such as the hypothalamus and hippocampus, accompanied by microglial activation and gliosis quantified by immunohistochemistry and cytokine mRNA/protein analysis, indicating sustained neuroinflammation due to obesity−associated inflammatory signaling (Schmitt and Gaspar, 2023). Moreover, obesity-associated neuroinflammation correlates positively with synaptic dysfunction, increased oxidative stress, and region-specific structural changes in the brain including enlarged ventricles and altered hippocampal gene expression, which are accompanied by measurable deficits in cognitive assays (i.e. memory and executive function tasks) in rodents (Rajput et al., 2025). Clinically, individuals with obesity exhibit elevated plasma levels of adipocyte-derived cytokines, which correlate with increased adiposity and systemic inflammation, indicating a sustained peripheral inflammatory state. Moreover, greater adiposity and inflammatory burden are associated with poorer cognitive performance and an estimated 20–40% increased risk of mild cognitive impairment and dementia later in life in these individuals. Collectively, these findings establish cytokine-driven metabolic inflammation as a critical mechanistic link between adipose tissue remodeling and central nervous system dysfunction in obesity.

### 4.2 Lipid mediators

In obesity, adipose tissue lipid mediator profiles shift toward increased cyclooxygenase (COX) and lipoxygenase (LOX) activity, with a significant increase in COX-2 and its enzyme Ptgs2 in the ATMs of DIO models (Schmitt and Gaspar, 2023). Enhanced COX-2 activity promotes the conversion of arachidonic acid into pro-inflammatory prostaglandins, particularly prostaglandin E_2_ (PGE_2_), which is elevated in inflamed adipose tissue and circulation in obese mice (Schmitt and Gaspar, 2023). Interestingly, inhibition of COX-2 reduces PGE_2_ levels and attenuates inflammatory gene expression, supporting a functional role for COX-derived lipid mediators in obesity-associated metabolic inflammation (Schmitt and Gaspar, 2023). Mechanistically, PGE_2_ binds EP receptors on macrophages, adipocytes, and microglial cells, triggering downstream cAMP–PKA and NF−κB signaling that enhances pro−inflammatory transcription (Mendes and Velloso, 2022). In HFD models, PGE_2_ signaling is correlated with central inflammatory signatures that include hypothalamic microglial activation and increased expression of IL−1β and TNFα, indicating that obesity−driven COX lipid mediators can influence CNS immune responses (Niraula et al., 2023). In parallel, LOX pathways contribute to pro−inflammatory mediator production. Adipose tissue from obese mice shows elevated 5−lipoxygenase (5-LOX) pathway enzyme expression and increased leukotriene production, with leukotrienes promoting macrophage recruitment and inflammatory cytokine expression (Horrillo et al., 2010). Leukotrienes act through Leukotriene B_4_ (BLT) and cysteinyl leukotriene (CysLT) receptors to activate MAPK and NF-κB signaling pathways, thereby amplifying pro-inflammatory gene expression in immune cells (Horrillo et al., 2010). Additionally, obesity alters levels of specialized pro−resolving lipid mediators that modulate immune responses via nuclear receptor pathways such as PPARγ, that are important for regulating glial and neuronal responses through transcriptional control (Liu X. et al., 2024). These findings indicate that dysregulated lipid mediator signaling in obesity amplifies systemic inflammation and extends beyond adipose tissue to modulate neurovascular integrity and glial activation within the CNS.

### 4.3 Extracellular vesicles lipids cargo

Beyond freely circulating lipid mediators, adipose tissue communicates with distant organs through extracellular vesicles (EVs), which transport bioactive lipids and other signaling molecules capable of modulating CNS function. In obesity, adipose tissue-derived EVs are enriched in lipid species such as lysophosphatidylcholine (LPC) and sphingomyelin, which have been shown to influence Aβ aggregation and neuronal uptake in experimental models (Yang et al., 2025). Functionally, small EVs (sEVs) isolated from the adipose tissue of individuals with obesity altered BBB integrity which was linked with decreased transendothelial electrical resistance (TEER) and tight junction protein expression indicating increased permeability (Mishra et al., 2026). This disruptive effect correlates with adiposity levels, suggesting that obesity-associated EVs contribute to BBB dysfunction and represent a mechanistic link between excess adipose tissue and adverse brain outcomes (Mishra et al., 2026). Moreover, adipose tissue-derived EVs isolated from individuals with obesity and insulin resistance were shown, upon transfer into recipient mice, to accumulate in hippocampal neurons and impair synaptic function leading to memory deficits, whereas selective depletion of pathogenic miRNAs from EVs attenuated these synaptic and cognitive impairments supporting a causal role for EV-mediated adipose-brain communication (Wang J. et al., 2022). Although direct *in vivo* evidence linking EV lipid cargo to microglial inflammatory activation remains limited, studies in related contexts suggest that alterations in EV composition can influence neural and endothelial cell phenotypes (De Paula et al., 2024). Collectively, these findings support a model in which obesity-associated changes in adipose-derived EV lipid cargo contribute to endothelial dysfunction, glial activation, and impaired neural signaling within the CNS.

## 5 Adipokines in adipose-brain axis

Adipokines such as leptin, adiponectin, and resistin constitute key endocrine signals linking adipose tissue to the CNS, where they integrate metabolic status with neural, inflammatory, and behavioral processes and are profoundly altered in obesity (**Table 1**).

**Table 1:** Major adipokines involved in adipose-brain communication.

Adipokine	Primary source	Receptors/pathways signaling	CNS access targets	Effects on the brain	Changes in obesity	Clinical/translational relevance
Leptin	WAT-adipocytes	LEP-R (class I cytokine receptor); JAK2/STAT3, JAK2/STAT5 signaling	• Crosses BBB and CSF • Act mainly on hypothalamus (ARC) neurons • Affects myeloid cells	• Suppresses appetite via inhibition of NPY • Regulates energy homeostasis • Modulates neuroimmune signaling	• Circulating levels increase with adiposity • Leptin resistance develops due to impaired BBB transport, reduced receptor expression, and disrupted signaling	• Limits efficacy of leptin therapy in obesity • Genetic leptin mutations linked to severe obesity • Leptin–STAT3 signaling contributes to adipose inflammation and insulin resistance
Adiponectin	Adipocytes	AdipoR1, AdipoR2; PPARα, CREB signaling; regulation of lipid metabolism	• Crosses BBB • Receptors expressed in cortex, hypothalamus, thalamus, hippocampus; neurons, microglia, astrocytes	• Neuroprotective and antidepressant effects • Regulate synaptic plasticity, lipid homeostasis, neuroinflammation, cognition, and behavior	• Circulating levels decline in obesity and T2DM • Associated with insulin resistance, neuroinflammation, and synaptic dysfunction	• AdipoRon reverses synaptic, inflammatory, and cognitive deficits in T2DM models • Potential therapeutic target for metabolic and neuroinflammatory disorders
Resistin	Rodents: WAT adipocytes; Humans: WAT-infiltrating macrophages	Putative receptors: TLR4, CAP1; activates MyD88/TIRAP, JNK, p38 MAPK, NF-κB pathways	• Produced in ARC and hippocampus. • Increases BBB permeability • Act on neurons, microglia, endothelial cells	• Promotes hypothalamic inflammation, insulin resistance, altered neuropeptide signaling • Increases oxidative stress and gliosis	• Circulating and hypothalamic levels increase in diet- induced obesity • Amplifies fatty acid– TLR4 signaling	• Contributes to early hypothalamic neuroinflammation • Implicated in AD pathology by promoting Aβ accumulation, oxidative stress, and cognitive decline

### 5.1 Leptin

Leptin is a key hormone involved in long-term regulation of energy balance and appetite and signals through its transmembrane receptor, LEP-R, a member of the class I cytokine receptor family (Rhea et al., 2017; Rustenhoven et al., 2017). In circulation, leptin exists in free and protein-bound forms, with the free fraction considered biologically active (Sun et al., 2025). The LEP-R gene, located on chromosome 1p31, encodes multiple receptors that are found in adipocytes and endothelial cells (Mellott and Faulkner, 2023). Circulating leptin levels positively correlate with total adiposity and reflect whole body energy stores, decreasing during fasting and increasing with feeding and fat mass expansion (Obradovic et al., 2021). Leptin accesses the brain by crossing both the BBB and CSF interfaces, where it acts primarily on hypothalamic circuits to suppress food intake and promote energy expenditure through a negative feedback loop between adipose tissue and the CNS (Obradovic et al., 2021). Beyond its role in energy homeostasis, leptin exerts pleiotropic effects on neuroendocrine and immune functions, consistent with its cytokine-like properties. In obesity, chronically elevated leptin levels are associated with leptin resistance, resulting in impaired central leptin signaling and contributing to dysregulated metabolic and inflammatory responses (Obradovic et al., 2021). This leads to reduced leptin transport to target cells, decreased LEP-R expression, and disruptions in downstream signaling (Wang D. et al., 2022). Consequently, leptin resistance not only promotes the development and persistence of obesity but also limits the therapeutic effectiveness of exogenous leptin (Olivier et al., 2000). A recent study demonstrates that circulating levels of the hepatokine Fetuin B are positively associated with leptin in obese individuals and diet induced obese mice, and that leptin directly upregulates Fetuin B transcription and expression in hepatocytes via a STAT3 dependent signaling pathway (Wang D. et al., 2022). Furthermore, Fetuin B partially mediates the effect of leptin resistance in obesity, suggesting a mechanistic link between adipose derived leptin and metabolic dysfunction through an adipose–liver axis (Wang D. et al., 2022). In obese CD-1 mice, the ability of circulating leptin to cross the BBB becomes progressively impaired as obesity develops, even though lean mice maintain normal leptin transport rates (Banks and Farrell, 2003). This impairment is not innate but acquired with obesity and can be reversed by modest weight reduction, indicating that leptin resistance at the BBB is a dynamic process linked to body weight rather than a fixed defect (Banks and Farrell, 2003). A recent clinical study further suggested that a normal weight insulin resistant phenotype may be associated with elevated plasma leptin concentrations and a higher leptin to fat mass ratio in young women (Minato-Inokawa et al., 2023). Interestingly, a rare nonsynonymous mutation in the leptin gene (H118L) was identified in a clinical cohort of severely obese individuals which was absent in lean controls suggesting a potential genetic contributor to extreme adiposity (Zhao et al., 2014). Although rare and requires further functional validation, this finding supports the concept that monogenic alterations in leptin can be associated with severe obesity in humans (Zhao et al., 2014).

Leptin is known for regulating appetite and metabolism by inhibiting the synthesis and release of neuropeptide Y (NPY) in the arcuate nucleus (ARC), acting on both the satiety and hunger centers of the hypothalamus (Stephens et al., 1995). It exerts its effects by activating the Janus kinase 2 (JAK2) receptor in the hypothalamus. This activation triggers a phosphorylation cascade within the intracellular domain of LEP-R, enabling the recruitment of signal transducers and activators of transcription (STAT) proteins (St-Pierre and Tremblay, 2012). Depending on the phosphorylated residue, different signaling pathways are engaged; phosphorylation of Y1077 activates the JAK2/STAT5 pathway, which helps prevent obesity, while phosphorylation of Y1138 triggers the JAK2/STAT3 pathway, essential for maintaining energy homeostasis (St-Pierre and Tremblay, 2012; Xiong et al., 2024). However, under HFD induced obesity JAK2/STAT3 signaling in myeloid cells promotes adipose tissue inflammation and insulin resistance in mice (Zhang C. et al., 2025). Myeloid-specific deletion of JAK2 or STAT3 reduces adipose tissue macrophage infiltration, dampens pro-inflammatory M1 cytokine production induced by free fatty acids, and improves metabolic outcomes, while adoptive transfer of JAK2-deficient myeloid cells or *in vivo* STAT3 silencing attenuates obesity associated inflammation (Zhang C. et al., 2025). Since leptin dysregulation in obesity has long been linked to central resistance and impaired energy homeostasis, emerging research highlights the complementary role of adiponectin as an anti-inflammatory adipokine whose levels are often reduced in obesity and inversely associated with metabolic dysfunction (Tremblay et al., 2024). This shift underscores a transition from leptin-focused to adiponectin-centered mechanisms in adipose brain communication and systemic metabolic regulation (Tremblay et al., 2024).

### 5.2 Adiponectin

Adiponectin is a polypeptide hormone belonging to the complement C1q family that is predominantly secreted by adipocytes (Rizzo et al., 2020). In the periphery, adiponectin plays a central role in metabolic homeostasis by enhancing insulin sensitivity, reducing oxidative stress, and exerting anti-inflammatory effects through activation of its receptors, AdipoR1 and AdipoR2 (Rizzo et al., 2020). Circulating adiponectin levels are inversely correlated with adiposity and are reduced in obesity and metabolic disease, contributing to systemic insulin resistance and chronic inflammation (Pan and Kastin, 2007). It can access the brain from the circulation by crossing the BBB, where it exerts neuroprotective and antidepressant effects (Pan and Kastin, 2007). Beyond these peripheral functions, growing evidence shows that adiponectin plays an important regulatory role in brain inflammation (Kadowaki et al., 2008). AdipoR1 and AdipoR2 are widely expressed in different brain regions such as the cortex, hypothalamus, thalamus and hippocampus and is found mainly in neurons, microglia, and astrocytes, allowing adiponectin to modulate several neuroimmune pathways (Clain et al., 2022). Functional studies support a critical role for adiponectin signaling in neuronal integrity and behavior. Knockdown of hippocampal AdipoR1 via adeno-associated virus (AAV) mediated stereotaxic delivery induces depressive-like behavior and impairs hippocampal synaptic plasticity by reducing dendritic spine density and altering excitatory/inhibitory (E/I) synapse balance (Zhu P. et al., 2025). Similarly, AdipoR2-deficient mice exhibit increased brain weight, reduced cellular density, and marked alterations in membrane lipid composition driven by elevated palmitic acid and reduced oleic acid levels, resulting in lipotoxic stress (Ruiz et al., 2024). These findings indicate that AdipoR2 is essential for sensing excess saturated fatty acids and maintaining lipid homeostasis within the brain, and that its loss disrupts neuronal membrane integrity and function (Ruiz et al., 2024). Consistent with these structural alterations, AdipoR2-deficient mice also display anxiety-like behavior and hyperactivity, further linking adiponectin signaling to lipid metabolism and neural function (Ruiz et al., 2024). Importantly, reduced adiponectin levels are associated with increased age-related neuroinflammation in both mice and humans (He et al., 2023). Adiponectin-deficient mice develop age-dependent cognitive decline and emotional dysregulation, underscoring a critical role for adiponectin in preserving brain homeostasis and limiting neuroinflammatory processes across the lifespan (He et al., 2023).

In obesity, circulating adiponectin levels decline significantly leading to insulin resistance and hyperinsulinemia (Nigro et al., 2014). In a T2DM mouse model (HFD combined with intraperitoneal streptozotocin injection), downregulation of AdipoR2 in the hippocampus was associated with reduced expression of postsynaptic proteins such as PSD95 and GluA1, leading to impaired cognitive performance (Gong et al., 2026). These mice also showed decreased phosphorylation of PPARα and CREB, which correlated with increased astrogliosis and microgliosis in the hippocampus (Gong et al., 2026). Notably, all these dysfunctions were reversed following administration of AdipoRon, an adiponectin receptor agonist, which restored synaptic protein expression, normalized signaling pathways, reduced glial activation, and improved behavioral outcomes in the treated mice (Gong et al., 2026). Furthermore, intracerebroventricular (i.c.v) administration of adiponectin in mice has been shown to decrease body weight independently of food intake by increasing energy expenditure and stimulating thermogenesis (Qi et al., 2004). Moreover, exposing adiponectin knock-out (ADP-KO) mice to HFD led to dyslipidemia and hyperglycemia, and these mice displayed prolonged seizure activity following low-dose kainic acid administration. They also had increased astroglial and microglial activation, together with neuronal damage in the hippocampus, underscoring adiponectin’s involvement in seizure-related neuropathology (Lee et al., 2011). Overall, the evidence highlights adiponectin as an essential neuroprotective adipokine whose decline in obesity disrupts lipid homeostasis, amplifies neuroinflammation, and compromises synaptic and behavioral functions.

### 5.3 Resistin

Resistin is a pro-inflammatory adipokine originally identified as a link between obesity and insulin resistance. In rodents, resistin is predominantly produced by WAT adipocytes, whereas in humans it is mainly secreted by macrophages infiltrating WAT, highlighting important species-specific differences in its cellular origin (Steppan et al., 2001). Its expression is upregulated during adipogenesis, with levels increasing throughout adipocyte differentiation, and circulating resistin concentrations are elevated in obesity, promoting insulin resistance and imparing glucose tolerance (Delle et al., 2023). In parallel, resistin production is closely linked to inflammatory activation of myeloid cells, supporting its role as a metabolic mediator rather than a classical adipocyte-derived hormone (Steppan et al., 2001). Beyond its peripheral metabolic effects, resistin contributes to neurovascular dysfunction by promoting endothelial inflammation and increasing BBB permeability. These effects are associated with reduced expression of tight junction proteins, including ZO-1 and occludin-1, and are mediated by oxidative stress and activation of the p38/MAPK signaling pathway (Jamaluddin et al., 2013; Takechi et al., 2017). Resistin expression has also been detected in several brain regions, including the ARC and hippocampus, suggesting potential local actions within the CNS (Badoer, 2021). Functional studies support a direct role for central resistin in hypothalamic inflammation and metabolic regulation. Knockdown of the hypothalamic short resistin isoform (s-resistin) markedly reduced hypothalamic inflammation and enhanced insulin and leptin signaling in mice (Rodríguez et al., 2018). This intervention lowered the expression of pro-inflammatory mediators such as TNF-α, IL-6, and iNOS, reduced activation of JNK and NF-κB pathways, and normalized appetite-regulating neuropeptides, including NPY and Proopiomelanocortin (POMC). These findings identify endogenous hypothalamic resistin as a key driver of neuroinflammation and whole-body insulin resistance (Rodríguez et al., 2018). Despite its discovery more than two decades ago, the canonical resistin receptor has not yet been identified. Current evidence suggests that adenyl cyclase–associated protein-1 (CAP1), which mediates monocyte inflammatory responses, and TLR4 may function as resistin-responsive receptors, thereby linking resistin signaling to innate immune and inflammatory pathways (Badoer, 2021).

Experimental evidence indicates that disruptions in lipid storage generate hormonal and nutrient-derived signals capable of influencing specific regions of the CNS and promoting neuroinflammation. This process is driven in part by the activation of TLR4 through circulating fatty acids (FAs), which are transported bound to albumin, allowing them to cross the BBB and enter the brain parenchyma, where they alter food intake and metabolic regulation (Lam et al., 2005). Studies also highlighted the neuroinflammatory effect of TLR4 on the brain microglia and macrophages, and its absence improved cognition, hypothalamic functions, and metabolism (Zhou et al., 2020; Liu J. et al., 2024). Given the strong interaction between resistin and TLR4 signaling, resistin may act as a critical amplifier of obesity-induced neuroinflammation. A study demonstrated that administering resistin into a rat’s brain via i.c.v. infusion dysregulated energy homeostasis, insulin sensitivity and blood glucose levels in the hypothalamus (Benomar et al., 2013). The mechanism behind this is that resistin binds directly to TLR4 on neurons, activating MyD88/TIRAP-dependent inflammatory pathways (i.e. JNK and p38 MAPK) that inhibit IRS-1 and insulin receptor signaling. Through this mechanism, resistin promotes hypothalamic inflammation and widespread insulin resistance in the body (Benomar et al., 2013). Recent evidence further identifies a resistin-dependent mechanism driving early hypothalamic neuroinflammation. HFD feeding increases resistin expression in the mediobasal hypothalamus, where resistin activates TLR4 and induces the pro-inflammatory microRNA miR-155-5p through NF-κB, JNK, and p38 MAPK signaling (Prévost et al., 2025). Both resistin and palmitate elevated miR-155-5p in microglial cells, indicating convergence between adipokine and saturated fatty acid signaling. Increased miR-155-5p suppressed targets such as Quaking and Elmo1, impairing microglial function (Prévost et al., 2025). Notably, knocking down miR-155-5p in the hypothalamus improved glucose tolerance and restored microglial gene expression. These findings highlight a novel resistin/TLR4/miR-155-5p axis that contributes to the initiation of hypothalamic neuroinflammation and early metabolic dysregulation during obesity (Prévost et al., 2025).

Accumulating evidence suggests that resistin contributes to the progression of neurodegenerative diseases since it has a proinflammatory role. A case control study found that plasma resistin concentrations were lower in patients with AD and it was correlated with an increase in Aβ_1-42_ concentration in the CSF (Marcinnò et al., 2022). Supporting this finding, inducing metabolic syndrome in AD transgenic mouse model (APP/PSNI) showed that excess resistin worsens Aβ accumulation, increases oxidative stress, impairs glucose utilization and accelerates cognitive decline (Cisternas et al., 2023). This is observed in the hippocampus and cortex and is linked with increased gliosis and lipid peroxidation (higher 4-HNE) (Cisternas et al., 2023). This causal evidence from *in vivo* models strengthens the argument that resistin dysregulation could actively contribute to neurodegenerative processes, rather than simply reflecting an epiphenomenon of metabolic dysfunction. Overall, resistin acts as a critical inflammatory signal connecting adipose dysfunction to both metabolic and neurodegenerative impairment in the brain.

## 6 Sex differences in adiposity-associated inflammation

Males and females exhibit distinct physiological responses to adiposity, leading to marked sex differences in the prevalence and severity of obesity-associated diseases (Costa et al., 2025). These disparities arise from variations in sex hormones, fat distribution, immune cell composition, and adipokine profiles, all of which shape inflammatory responses during obesity (Singer et al., 2015; Costa et al., 2025). Recent evidence shows that sex differences in fat distribution play a critical role in shaping inflammatory outcomes during obesity (Stranahan et al., 2023). Female mice display a more protective subcutaneous-to-visceral fat ratio than males, which limits adipose inflammation (Stranahan et al., 2023). Moreover, male mice exhibited increased macrophage infiltration from the bone marrow to the brain parenchyma along with upregulation of TLR4, all which was delayed in female mice. Notably, surgically reducing subcutaneous fat in females abolishes this protection and results in inflammation levels comparable to males (Stranahan et al., 2023). Another study showed that male mice under HFD accumulate significantly more pro-inflammatory CD11c^+^ adipose macrophages than females (Singer et al., 2015). This is because male hematopoietic progenitor cells generate more inflammatory monocytes and their adipose tissue recruits them more strongly under metabolic conditions compared to females (Singer et al., 2015). Consistent with these findings, a recent clinical study demonstrated that men with obesity have markedly higher adipose tissue insulin resistance than women, driven by impaired insulin-mediated suppression of lipolysis and lower Insulin receptor substrate 1 (IRS1) expression in male adipocytes (Arner et al., 2024). This increased adipose insulin resistance promotes elevated circulating fatty acids and may further amplify systemic and tissue-specific inflammation in males (Arner et al., 2024). Importantly, these sex-specific differences extend to cognitive outcomes, although vulnerability appears context dependent. For example, HFD adminstration to 3xTg-AD mouse model (a triple-transgenic Alzheimer’s disease model that develops both amyloid-β and tau pathology) exacerbates hippocampal neuroinflammation and cognitive decline in female mice compared with males (Gannon et al., 2022), whereas cerebrovascular dysfunction and cognitive impairment are more pronounced in males in diet-induced obesity models, suggesting that sex influences the dominant pathological pathway linking obesity to cognitive decline (Yen et al., 2025). Altogether, these studies demonstrate that adipose tissue behavior is highly sex-dependent, shaping both local and systemic inflammatory responses to metabolic stress.

## 7 Therapeutic approaches

### 7.1 Lifestyle interventions

Lifestyle modification remains the cornerstone of obesity prevention and management, with diet and physical activity representing the most effective non-pharmacological strategies to limit excessive adiposity (**Table 2**) (Kolnes et al., 2021). An 8-week supervised aerobic exercise intervention in sedentary adults who are overweight or obese was shown to enhance brain insulin sensitivity, increasing insulin-induced neural activity in striatal regions and strengthening functional connectivity in hippocampal networks to levels comparable with healthy weight individuals (Kullmann et al., 2022). Interestingly, a systematic review and meta-analysis of nearly 4, 000 adults with overweight and obesity found that dietary interventions were more effective than exercise alone at decreasing circulating leptin and increasing adiponectin, thus regulating energy balance and metabolism (Khalafi et al., 2023), Moreover, individuals with mild cognitive impairment and AD exhibited lower circulating levels of leptin but not adiponectin compared to those with cognitive decline, highlighting how adipose derived hormones can reflect metabolic and neurological status tied to excess adiposity (Carbone et al., 2024). Experimentally, in a rat model of T2DM, high-intensity interval training (HIIT) reversed diabetes induced impairments in hippocampal insulin and adiponectin signaling, restored beneficial kinase activity, and reduced the accumulation of tau protein changes that align with improved metabolic and neuroprotective profiles (Khoramipour et al., 2023). Furthermore, applying endurance treadmill exercise on 8-month-old rats under HFD markedly attenuated HFD induced hippocampal neuroinflammation by suppressing TLR4–MyD88–NF-κB signaling, leading to reduced expression of pro-inflammatory cytokines (TNF-α, IL-1β, COX-2) and decreased microglial and astrocytic activation (Kang et al., 2016). Importantly, these anti-inflammatory effects were accompanied by improved insulin sensitivity, reduced visceral adiposity, prevention of neuronal apoptosis, and restoration of hippocampal-dependent working memory (Kang et al., 2016). In HFD induced obese mice, 12 weeks of treadmill exercise alleviated cognitive impairment by enhancing hippocampal neuroplasticity, significantly increasing expression of brain derived neurotrophic factor (BDNF) and its receptor TrkB compared with sedentary obese controls (Kim et al., 2016). Moreover, HFD exposure initiated during adolescence in mice impaired hippocampus dependent flexible memory and reduced the number of immature neurons in the dentate gyrus, effects that were fully prevented by long-term voluntary physical exercise using a running wheel (Klein et al., 2016). Furthermore, exercise mediated preservation of cognitive flexibility was associated with enhanced adult hippocampal neurogenesis and increased survival of newborn mature neurons, highlighting physical activity as a potent modulator of obesity related brain dysfunction during vulnerable developmental windows (Klein et al., 2016).

**Table 2:** Summary of therapeutic approaches targeting the adipose–brain axis in obesity.

Therapeutic strategy	Primary target	Adipose and metabolic effects	CNS effects	Key mechanisms	Evidence type	Key outcomes
Dietary intervention (caloric restriction; IER; Mediterranean; ketogenic)	Energy balance; macronutrient composition	• ↓ adiposity and leptin • ↑ adiponectin and improved insulin sensitivity and lipid profile	• Improved hippocampal-dependent memory • ↑ cortical thickness in AD-vulnerable regions • Favorable CSF Aβ/tau shifts (diet- dependent)	• Reduced adipose inflammation and normalized adipokines • Modulation of cerebrovascular conductance • Reduced oxidative stress and microglial activation	Meta-analysis; RCTs; rodent HFD models	• More effective than exercise alone in regulating adipokines • Reshapes neural circuits regulating reward and cognition
Aerobic exercise	Increased energy expenditure; improved insulin action	• ↓ visceral fat and improved glucose metabolism	• Enhanced brain insulin sensitivity • ↑ striatal insulin-induced activity and restored hippocampal connectivity	• Improved central insulin signaling and reduced metabolic inflammation	Human intervention	• Restores neural activity to lean-like levels
HIIT	High-intensity metabolic stress adaptation	• Improved insulin sensitivity and reduced hyperglycemia	• Restored hippocampal insulin/adiponectin signaling • ↓ tau pathology	• Activation of neuroprotective kinase pathways and restored insulin–Akt signaling	Rodent T2DM models	• Reverses diabetes-induced cognitive and signaling impairments
Endurance/treadmill exercise	Sustained aerobic activity	• ↓ visceral adiposity and improved metabolic profile	• Reduced neuroinflammation • Improved memory and neuroplasticity (↑ BDNF/TrkB)	• Suppression of TLR4–MyD88–NF-κB signaling • ↓ TNF-α, IL-1β; ↓ microgliosis and astrogliosis	Rodent HFD models	• Prevents neuronal apoptosis • Restores working memory
Voluntary physical activity (adolescence)	Long-term spontaneous exercise	• Protection against HFD- induced metabolic stress	• Preserved cognitive flexibility • ↑ hippocampal neurogenesis	• Enhanced survival of newborn neurons and developmental neuroplasticity	Rodent developmental models	• Prevents adolescent HFD- induced cognitive impairment
Omega-3 PUFA supplementation (EPA/DHA)	Lipid composition; inflammatory modulation	• Improved glucose tolerance and restored anti-inflammatory lipid profile	• Reduced gliosis (↓ GFAP, Iba-1) • Improved hippocampal memory	• ↑ EPA: AA ratio • Reduced neuroinflammation • Preserved gray matter and connectivity (early-life exposure)	Rodent models; RCTs	• Neuroprotection depends on baseline omega-3 status and developmental timing
Orlistat	Gastric/pancreatic lipase inhibition	• ↓ fat absorption and adiposity • Improved lipid profile	• Reduced hippocampal inflammation • Improved recognition memory	• Indirect CNS effects via reduced metabolic stress • ↓ TNF-α, IL-1β restored oxidative balance	Rodent models; RCTs	• Peripheral action with secondary brain benefits
GLP-1 receptor agonists (e.g., semaglutide)	Central & peripheral GLP-1R activation	• ↓ body weight and visceral adipocyte size • Improved glycemic control	• Improved BBB and neurovascular integrity • ↓ leukocyte-endothelium interactions • ↓ microglial activation and improved cognition and motor function	• Enhanced satiety signaling and neurovascular protection • NF-κB suppression, reduced oxidative stress and anti-apoptotic signaling	Preclinical; early clinical trials; ongoing Phase III (AD/PD)	• Benefits extend beyond weight loss to neurovascular and neuroimmune modulation
Dual GLP-1/GIP agonists (tirzepatide)	Combined incretin receptor activation	• Weight loss • ↑ fat oxidation & thermogenesis	• Altered neural food-cue responses • Restored synaptic proteins • ↓ microglial activation and improved motor and cognitive outcomes in PD/T2DM models	• Inhibition of NLRP3 inflammasome • Restoration of PI3K/Akt/GSK3β signaling	Clinical trials; multiple rodent neurodegenerative models	• Enhanced efficacy via central- peripheral synergy • Robust preclinical neuroprotection
Triple GLP-1/GIP/Glucagon agonist (retatrutide)	Multi-incretin receptor activation	• Weight loss • Improved insulin sensitivity and enhanced fat oxidation and energy expenditure	• CNS effects not yet fully characterized; potential central modulation inferred from incretin receptor expression and multi- agonist preclinical data	• Likely combined satiety signaling, metabolic anti-inflammatory effects, and possible neuroimmune modulation (preclinical evidence emerging)	Phase II clinical trials (metabolic); limited preclinical CNS data	• Promising metabolic efficacy • Direct neuroprotective effects remain to be established

↑ indicates increase/upregulation; ↓ indicates decrease/downregulation.

Beyond physical activity, targeted nutritional interventions represent an equally powerful strategy to modulate obesity-associated metabolic dysfunction and its downstream effects on the brain. Intermittent calorie restriction has emerged as a particularly promising approach. In adults with central obesity, a four-week energy-restricted diet significantly improved hippocampal-dependent memory performance, accompanied by increased circulating biomarkers linked to neurogenesis, including BDNF, alongside reductions in body weight and adiposity, suggesting enhanced hippocampal plasticity through neurotrophic and metabolic modulation (Kim et al., 2020). Similarly, in obese adults, a two-month intermittent calorie intervention altered resting-state brain activity by increasing regional homogeneity in visual attention and sensorimotor regions (e.g. lingual gyrus, calcarine cortex) while decreasing activity in other networks. These neural adaptations correlated with improved eating behavior control, weight loss, and favorable shifts in adipokines and metabolic markers, indicating that IER reshapes neural circuits involved in reward and self-regulation (Li et al., 2023). Complementing these clinical findings, HFD mice subjected to intermittent fasting exhibited improved cognitive performance, reduced neuroinflammation (lower lipocalin-2 and galectin-3 expression), decreased microglial activation, reduced hippocampal oxidative stress, and enhanced synaptic plasticity markers, together with improved metabolic profiles, highlighting reductions in adiposity and systemic inflammation as key mediators of fasting-induced neuroprotection (Lee et al., 2024). Ketogenic and Mediterranean-style dietary strategies further underscore the importance of macronutrient composition in shaping neurobiological outcomes. In older adults at risk for AD, reductions in visceral adiposity during a modified Mediterranean ketogenic diet were associated with beneficial shifts in CSF Aβ biomarkers, whereas fat loss during a low-fat diet correlated with changes in tau and cholinesterase activity, suggesting that ketogenic-based interventions may differentially influence neurodegenerative biomarker profiles (Brinkley et al., 2022). Moreover, greater adherence to a Mediterranean diet in adults with overweight and obesity was associated with increased cortical thickness in AD vulnerable regions such as the entorhinal cortex and precuneus, as well as improved executive function and memory, with benefits correlating with lower systemic inflammation and improved insulin sensitivity and lipid profiles (Kackley et al., 2022). Supporting a mechanistic role for ketosis, short-term β-hydroxybutyrate (ketone monoester) supplementation in adults with obesity significantly increased CSF and cerebrovascular conductance in major arteries and improved complex cognitive task performance as improvements in working memory were positively correlated with enhanced cerebrovascular function, suggesting that exogenous ketosis may support cognition through vascular mechanisms independent of circulating BDNF changes (Walsh et al., 2021).

Finally, omega-3 polyunsaturated fatty acid supplementation demonstrates context-dependent neuroprotective potential (Layé et al., 2018). In diet-induced obese mice, EPA/DHA-rich fish oil improved glucose tolerance, reduced hyperphagia, and alleviated anxiety- and depressive-like behaviors without altering body weight. This is accompanied by restoration of anti-inflammatory brain lipid profiles (increased EPA, DPA, DGLA; reduced arachidonic acid), an elevated EPA: AA ratio, and reduced forebrain gliosis markers (GFAP and Iba-1), indicating attenuation of neuroinflammation (Demers et al., 2020). In contrast, an 18-week randomized placebo-controlled trial in healthy mid-life adults with low dietary omega-3 intake found no significant improvement in global cognition or brain morphology following EPA/DHA supplementation, although modest executive function benefits were observed in individuals with particularly low baseline DHA levels, suggesting that cognitive responsiveness may depend on pre-existing omega-3 status (Leckie et al., 2020). Importantly, in ApoE-Leiden mice, early-life supplementation with long-chain polyunsaturated fatty acids (ARA and DHA) mitigated high-fat/high-carbohydrate diet induced metabolic dysfunction, preserved gray matter integrity and functional connectivity, improved hippocampal dependent memory, and counteracted cerebrovascular impairments later in life, underscoring the developmental timing-dependent neurovascular and structural resilience conferred by early lipid interventions (Arnoldussen et al., 2016). Collectively, these findings indicate that dietary interventions targeting energy balance, macronutrient composition, and lipid quality can modulate systemic inflammation, adiposity, cerebrovascular function, and neuroplasticity, thereby offering mechanistically grounded strategies to attenuate obesity-associated brain vulnerability.

### 7.2 Pharmacological treatments

Most weight loss pharmacological treatments act centrally to enhance satiety and reduce food intake, thereby supporting adherence to dietary interventions with the primary goal of sustained weight reduction (Salas-Venegas et al., 2022). Current clinical guidelines recommend several drug classes for obesity management, many of which target neuroendocrine pathways regulating appetite and energy homeostasis (**Table 2**). Among these, orlistat represents a peripherally acting anti-obesity drug that promotes weight loss by inhibiting gastric and pancreatic lipases, reducing intestinal fat absorption by up to ~30% and limiting lipid storage in adipose tissue (Hollander, 2003). This decrease in dietary fat uptake leads to reduced adiposity and improvements in insulin sensitivity, lipid profiles, and other components of metabolic syndrome (Hollander, 2003). Because orlistat exhibits minimal systemic absorption, it does not directly target the brain; instead, it indirectly influences central energy balance by lowering circulating lipids and adiposity-associated metabolic stress, thereby mitigating obesity-related risk factors that affect brain function and metabolic neuroimmune interactions (Hollander, 2003). In line with this concept, administrating orlistat to HFD-induced obese rats reduced hippocampal inflammation and oxidative stress, as indicated by decreased TNF-α and IL-1β levels and restoration of serum thiol-disulfide balance, accompanied by improvements in recognition memory and spatial learning comparable to lean controls (Yigit et al., 2024). Clinically, a 54-week randomized controlled trial in obese adolescents demonstrated that orlistat, combined with lifestyle intervention, produced modest but significant reductions in BMI, waist circumference, and fat mass compared with placebo, with an acceptable safety profile despite increased gastrointestinal side effects (Chanoine et al., 2005).

Beyond peripheral approaches, intestinal hormones have emerged as key therapeutic targets due to their central roles in energy homeostasis and satiety signaling (Geloneze et al., 2017). Among these, glucagon-like peptide-1 (GLP-1), an L-cell derived peptide, has gained prominence following the clinical success of GLP-1 receptor agonists in the treatment of diabetes and obesity. GLP-1 receptor (GLP-1R) signaling enhances glucose-dependent insulin secretion and exerts broad metabolic, cardiovascular, immune, and neurophysiological effects that collectively regulate appetite, energy expenditure, and body weight (Geloneze et al., 2017). In a diet-induced metabolic syndrome mouse model, chronic semaglutide treatment improved systemic metabolic parameters and reduced visceral adipocyte size while restoring neurovascular unit integrity, as evidenced by reversal of cerebral microvascular rarefaction and increased astrocytic coverage of brain capillaries (Estato et al., 2025). Although microglial activation was not fully reversed, semaglutide reduced leukocyte-endothelium interactions in cerebral vessels, supporting a protective effect on BBB integrity and cerebral microcirculation in metabolic disease (Estato et al., 2025). Another study further demonstrated that administrating semaglutide to diet-induced rodents reduces their food intake, alters food preference, and induces significant weight loss without reducing energy expenditure, acting through GLP-1 receptors in distributed brain regions, including the hypothalamus and hindbrain, to modulate neural circuits controlling feeding behavior, reward processing, and energy balance (Gabery et al., 2020). In the context of neuroinflammation, GLP-1 receptor agonists exert direct neuroprotective effects across metabolic and neurodegenerative models. Administration of semaglutide to T2DM rats with PD reduced oxidative stress, downregulated GFAP and caspase-3 expression, and restored Nrf2 signaling, leading to reduced cytokine production and neuronal injury (Salem et al., 2025). Similarly, treatment with the GLP-1 receptor agonist exendin-4 in HFD mice decreased microglial activation and reduced hippocampal TNF-α and IL-1β levels, accompanied by suppression of NF-κB signaling and improvements in synaptic plasticity and cognitive performance (Zhu Y. et al., 2025). This is further supported by another study where they also observed that semaglutide treatment to T2DM mice enhances cognitive performance, preserve hippocampal structure and reduce neuronal apoptosis (Zhu Y. et al., 2025). In addition to preclinical data, GLP-1 receptor agonists have shown neuroprotective effects in early clinical trials, with improvements in motor function in PD patients and cognitive outcomes in AD, and ongoing Phase III trials are evaluating semaglutide for these indications, highlighting the translational potential of GLP-1 signaling in human CNS disorders (Hölscher, 2024).

More recently, tirzepatide, a dual GLP-1 and GIP receptor agonist, has shown enhanced efficacy through combined peripheral and central mechanisms (Martin et al., 2025). In a 6-week randomized Phase 1 clinical trial in adults with overweight or obesity, tirzepatide markedly reduced energy intake and appetite measures compared with placebo and liraglutide, with functional brain imaging revealing altered neural responses to food cues, indicating engagement of central pathways regulating ingestive behavior (Martin et al., 2025). Extending these findings mechanistically, preclinical studies demonstrated that central administration of tirzepatide into mice under HFD induces weight loss independent of caloric intake by shifting substrate utilization toward fat oxidation (Zhang A. et al., 2025). This effect is mediated by enhanced lipolysis and thermogenesis in WAT and BAT via sympathetic nervous system signaling originating from hypothalamic and hindbrain nuclei, establishing a CNS-adipose tissue axis as a key driver of tirzepatide’s anti-obesity effects (Zhang A. et al., 2025). Moreover, tirzepatide exerts robust neuroprotective effects across metabolic and neurodegenerative models. In a rotenone-induced rat model of PD, tirzepatide restored motor function, increased striatal dopamine levels, reduced oxidative stress and pro-inflammatory cytokines (TNF-α, IL-6), and decreased α-synuclein hyperphosphorylation, while preserving substantia nigra neuronal integrity in a dose-dependent manner, with greater efficacy than exendin-4 (Delvadia et al., 2025). Similarly, mice under HFD had improved cognitive performance, restored synaptic protein expression, and attenuated microglial activation and neuroinflammation, effects mechanistically linked to SIRT3 upregulation, suppression of oxidative stress, and inhibition of NLRP3 inflammasome activation upon tirzepatide administration (Ma et al., 2024). Complementing these findings, treating diabetic rats with tirzepatide enhanced their spatial learning and memory, promoted dendritic spine formation, reduced Aβ accumulation and hippocampal structural damage, and normalized inflammatory signaling via restoration of the PI3K/Akt/GSK3β pathway, highlighting its capacity to modulate insulin resistance–associated neuroinflammatory and synaptic dysfunction (Guo et al., 2023).

## 8 Perspectives and future work in the field

Obesity is increasingly recognized as a state of systemic and central inflammation, in which adipose tissue functions as an active endocrine and immune organ capable of reshaping brain physiology. Adipose–brain communication operates through adipokines, inflammatory mediators, metabolic substrates, and neural pathways that converge at key brain interfaces, including the BBB, perivascular spaces, and the glymphatic system. Disruption of these interfaces represents a critical mechanistic link between metabolic dysfunction and neuroinflammation. Despite being among the most immune-rich brain compartments, the meninges remain a largely unexplored site for sensing and integrating adipose-derived inflammatory signals during obesity, requiring focused investigation.

Leptin resistance and elevated resistin signaling remain central drivers of obesity-associated neurovascular and microglial inflammation. Defining cell-type-specific adipokine signaling pathways and identifying unresolved receptors, particularly resistin, are key priorities. In addition, sex differences critically shape adipose–brain interactions yet remain underrepresented in obesity research, underscoring the need to systematically incorporate sex as a biological variable. Finally, while lifestyle interventions and incretin-based therapies improve metabolic outcomes, their direct effects on brain inflammation and barrier integrity remain poorly characterized. The emergence of the triple receptor agonist retatrutide (GLP-1, GIP, and glucagon receptor agonist), which demonstrates superior metabolic efficacy compared with existing therapies, raises important questions regarding its impact on brain structure and function. Although preclinical studies of multi-incretin agonists, including retatrutide, suggest potential neuroprotective and anti-inflammatory effects in experimental models, these findings remain preliminary and are yet to be validated in clinical populations (Roy et al., 2025). Given the expression of GLP-1 and GIP receptors in appetite-regulating brain regions, future studies should determine whether such therapies can modulate central inflammatory and neuroimmune pathways. Integrating adipose-brain communication, barrier biology, and neuroimmune mechanisms will be essential to evaluate whether emerging obesity treatments can not only restore metabolic health but also prevent or reverse obesity-associated brain dysfunction.

## References

[B1] Ajuwon K. M. Spurlock M. E. (2005). Palmitate activates the NF-κB transcription factor and induces IL-6 and TNFα expression in 3T3-L1 adipocytes. J. Nutr. 135, 1841–1846. doi: 10.1093/jn/135.8.1841. PMID: 16046706

[B2] AL-Dalaeen A. AL-Domi H. (2022). Does obesity put your brain at risk? Diabetes Metab. Syndr. Clin. Res. Rev. 16, 102444. doi: 10.1016/j.dsx.2022.102444. PMID: 35247658

[B3] André C. Guzman-Quevedo O. Rey C. Rémus-Borel J. Clark S. Castellanos-Jankiewicz A. . (2017). Inhibiting microglia expansion prevents diet-induced hypothalamic and peripheral inflammation. Diabetes 66, 908–919. doi: 10.2337/db16-0586. PMID: 27903745

[B4] Arner P. Viguerie N. Massier L. Rydén M. Astrup A. Blaak E. . (2024). Sex differences in adipose insulin resistance are linked to obesity, lipolysis and insulin receptor substrate 1. Int. J. Obes. 48, 934–940. doi: 10.1038/s41366-024-01501-x. PMID: 38491191 PMC11217000

[B5] Arnoldussen I. A. C. Zerbi V. Wiesmann M. Noordman R. H. J. Bolijn S. Mutsaers M. P. C. . (2016). Early intake of long-chain polyunsaturated fatty acids preserves brain structure and function in diet-induced obesity. J. Nutr. Biochem. 30, 177–188. doi: 10.1016/j.jnutbio.2015.12.011. PMID: 27012634

[B6] Avgerinos K. I. Spyrou N. Mantzoros C. S. Dalamaga M. (2019). Obesity and cancer risk: Emerging biological mechanisms and perspectives. Metabolism 92, 121–135. doi: 10.1016/j.metabol.2018.11.001. PMID: 30445141

[B7] Badoer E. (2021). Cardiovascular and metabolic crosstalk in the brain: Leptin and resistin. Front. Physiol. 12. doi: 10.3389/fphys.2021.639417. PMID: 33679451 PMC7930826

[B8] Banks W. A. Farrell C. L. (2003). Impaired transport of leptin across the blood-brain barrier in obesity is acquired and reversible. Am. J. Physiol. Endocrinol. Metab. 285, E10–E15. doi: 10.1152/ajpendo.00468.2002. PMID: 12618361

[B9] Benomar Y. Gertler A. De Lacy P. Crépin D. Ould Hamouda H. Riffault L. . (2013). Central resistin overexposure induces insulin resistance through toll-like receptor 4. Diabetes 62, 102–114. doi: 10.2337/db12-0237. PMID: 22961082 PMC3526022

[B10] Brinkley T. E. Leng I. Register T. C. Neth B. J. Zetterberg H. Blennow K. . (2022). Changes in adiposity and cerebrospinal fluid biomarkers following a modified Mediterranean ketogenic diet in older adults at risk for Alzheimer’s disease. Front. Neurosci. 16. doi: 10.3389/fnins.2022.906539. PMID: 35720727 PMC9202553

[B11] Carbone G. Bencivenga L. Santoro M. A. De Lucia N. Palaia M. E. Ercolano E. . (2024). Impact of serum leptin and adiponectin levels on brain infarcts in patients with mild cognitive impairment and Alzheimer’s disease: a longitudinal analysis. Front. Endocrinol. 15. doi: 10.3389/fendo.2024.1389014. PMID: 38686200 PMC11056582

[B12] Chanoine J. P. Hampl S. Jensen C. Boldrin M. Hauptman J. (2005). Effect of orlistat on weight and body composition in obese adolescents: a randomized controlled trial. JAMA 293, 2873. doi: 10.1001/jama.293.23.2873. PMID: 15956632

[B13] Cisternas P. Gherardelli C. Gutierrez J. Salazar P. Mendez-Orellana C. Wong G. W. . (2023). Adiponectin and resistin modulate the progression of Alzheimer´s disease in a metabolic syndrome model. Front. Endocrinol. 14. doi: 10.3389/fendo.2023.1237796. PMID: 37732123 PMC10507329

[B14] Clain J. Couret D. Planesse C. Krejbich-Trotot P. Meilhac O. Lefebvre d’Hellencourt C. . (2022). Distribution of adiponectin receptors in the brain of adult mouse: Effect of a single dose of the adiponectin receptor agonist, AdipoRON, on ischemic stroke. Brain Sci. 12, 680. doi: 10.3390/brainsci12050680. PMID: 35625066 PMC9139333

[B15] Corvera S. (2021). Cellular heterogeneity in adipose tissues. Annu. Rev. Physiol. 83, 257–278. doi: 10.1146/annurev-physiol-031620-095446. PMID: 33566675 PMC8091658

[B16] Costa D. N. Santosa S. Jensen M. D. (2025). Sex differences in the metabolism of glucose and fatty acids by adipose tissue and skeletal muscle in humans. Physiol. Rev. 105, 897–934. doi: 10.1152/physrev.00008.2024. PMID: 39869194 PMC12139471

[B17] Cutugno G. Kyriakidou E. Nadjar A. (2024). Rethinking the role of microglia in obesity. Neuropharmacology 253, 109951. doi: 10.1016/j.neuropharm.2024.109951. PMID: 38615749

[B18] De A. Boleti A. De O. Cardoso P. Frihling B. F. E Silva P. . (2023). Adipose tissue, systematic inflammation, and neurodegenerative diseases. Neural Regener. Res. 18, 38. doi: 10.4103/1673-5374.343891. PMID: 35799506 PMC9241402

[B19] Delle C. Cankar N. Digebjerg Holgersson C. Hvorup Knudsen H. Schiøler Nielsen E. Kjaerby C. . (2023). Long-term high-fat diet increases glymphatic activity in the hypothalamus in mice. Sci. Rep. 13, 4137. doi: 10.1038/s41598-023-30630-y. PMID: 36914703 PMC10011420

[B20] Delvadia P. Dhote V. Mandloi A. S. Soni R. Shah J. (2025). Dual GLP-1 and GIP agonist tirzepatide exerted neuroprotective action in a Parkinson’s disease rat model. ACS Chem. Neurosci. 16, 818–825. doi: 10.1021/acschemneuro.4c00729. PMID: 39964252

[B21] Demers G. Roy J. Machuca-Parra A. I. Dashtehei Pour Z. Bairamian D. Daneault C. . (2020). Fish oil supplementation alleviates metabolic and anxiodepressive effects of diet-induced obesity and associated changes in brain lipid composition in mice. Int. J. Obes. 44, 1936–1945. doi: 10.1038/s41366-020-0623-6. PMID: 32546855

[B22] De Paula G. C. Aldana B. I. Battistella R. Fernández-Calle R. Bjure A. Lundgaard I. . (2024). Extracellular vesicles released from microglia after palmitate exposure impact brain function. J. Neuroinflammation 21, 173. doi: 10.1186/s12974-024-03168-7. PMID: 39014461 PMC11253458

[B23] Estato V. Obadia N. Chateaubriand P. H. Figueiredo V. Curty M. Costa Silva M. . (2025). Semaglutide restores astrocyte–vascular interactions and blood–brain barrier integrity in a model of diet-induced metabolic syndrome. Diabetol. Metab. Syndr. 17, 2. doi: 10.1186/s13098-024-01528-0. PMID: 39754250 PMC11699651

[B24] Feng Z. Fang C. Ma Y. Chang J. (2024). Obesity-induced blood-brain barrier dysfunction: Phenotypes and mechanisms. J. Neuroinflammation 21, 110. doi: 10.1186/s12974-024-03104-9. PMID: 38678254 PMC11056074

[B25] Fisk H. L. Childs C. E. Miles E. A. Ayres R. Noakes P. S. Paras-Chavez C. . (2022). Dysregulation of subcutaneous white adipose tissue inflammatory environment modelling in non-insulin resistant obesity and responses to omega-3 fatty acids – a double blind, randomised clinical trial. Front. Immunol. 13. doi: 10.3389/fimmu.2022.922654. PMID: 35958557 PMC9358040

[B26] Gabery S. Salinas C. G. Paulsen S. J. Ahnfelt-Rønne J. Alanentalo T. Baquero A. F. . (2020). Semaglutide lowers body weight in rodents via distributed neural pathways. JCI Insight 5, e133429, 133429. doi: 10.1172/jci.insight.133429. PMID: 32213703 PMC7213778

[B27] Gannon O. J. Robison L. S. Salinero A. E. Abi-Ghanem C. Mansour F. M. Kelly R. D. . (2022). High-fat diet exacerbates cognitive decline in mouse models of Alzheimer’s disease and mixed dementia in a sex-dependent manner. J. Neuroinflamm. 19, 110. doi: 10.1186/s12974-022-02466-2. PMID: 35568928 PMC9107741

[B28] Geloneze B. De Lima-Júnior J. C. Velloso L. A. (2017). Glucagon-like peptide-1 receptor agonists (GLP-1RAs) in the brain–adipocyte axis. Drugs 77, 493–503. doi: 10.1007/s40265-017-0706-4. PMID: 28233273 PMC5357258

[B29] Gong H. Liu Y. Xia L. Niu B. Kong J. Wang B. . (2026). Adiponectin signaling ameliorates cognitive dysfunction in type 2 diabetic mice by activating the hippocampal AdipoR2-PPARα/CREB pathway. Mol. Neurobiol. 63, 68. doi: 10.1007/s12035-025-05383-6. PMID: 41254427

[B30] Guillemot-Legris O. Muccioli G. G. (2017). Obesity-induced neuroinflammation: Beyond the hypothalamus. Trends Neurosci. 40, 237–253. doi: 10.1016/j.tins.2017.02.005. PMID: 28318543

[B31] Guo X. Lei M. Zhao J. Wu M. Ren Z. Yang X. . (2023). Tirzepatide ameliorates spatial learning and memory impairment through modulation of aberrant insulin resistance and inflammation response in diabetic rats. Front. Pharmacol. 14. doi: 10.3389/fphar.2023.1146960. PMID: 37701028 PMC10493299

[B32] He K. Nie L. Ali T. Liu Z. Li W. Gao R. . (2023). Adiponectin deficiency accelerates brain aging via mitochondria-associated neuroinflammation. Immun. Ageing 20, 15. doi: 10.1186/s12979-023-00339-7. PMID: 37005686 PMC10067304

[B33] Hollander P. (2003). Orlistat in the treatment of obesity. Prim Care Clin. Off. Pract. 30, 427–440. doi: 10.1016/S0095-4543(03)00042-3. PMID: 14567157

[B34] Hölscher C. (2024). Glucagon-like peptide-1 class drugs show clear protective effects in Parkinson’s and Alzheimer’s disease clinical trials: A revolution in the making? Neuropharmacology 253, 109952. doi: 10.1016/j.neuropharm.2024.109952. PMID: 38677445

[B35] Horrillo R. González-Périz A. Martínez-Clemente M. López-Parra M. Ferré N. Titos E. . (2010). 5-lipoxygenase activating protein signals adipose tissue inflammation and lipid dysfunction in experimental obesity. J. Immunol. 184, 3978–3987. doi: 10.4049/jimmunol.0901355. PMID: 20207999

[B36] Huang S. Rutkowsky J. M. Snodgrass R. G. Ono-Moore K. D. Schneider D. A. Newman J. W. . (2012). Saturated fatty acids activate TLR-mediated proinflammatory signaling pathways. J. Lipid Res. 53, 2002–2013. doi: 10.1194/jlr.D029546. PMID: 22766885 PMC3413240

[B37] Huang X. Wang Y. J. Xiang Y. (2022). Bidirectional communication between brain and visceral white adipose tissue: Its potential impact on Alzheimer’s disease. eBioMedicine 84, 104263. doi: 10.1016/j.ebiom.2022.104263. PMID: 36122553 PMC9490488

[B38] Jais A. Brüning J. C. (2017). Hypothalamic inflammation in obesity and metabolic disease. J. Clin. Invest 127, 24–32. doi: 10.1172/JCI88878. PMID: 28045396 PMC5199695

[B39] Jamaluddin M. S. Yan S. Lü J. Liang Z. Yao Q. Chen C. (2013). Resistin increases monolayer permeability of human coronary artery endothelial cells. PloS One 8, e84576. doi: 10.1371/journal.pone.0084576. PMID: 24386395 PMC3874001

[B40] Kackley M. L. Brownlow M. L. Buga A. Crabtree C. D. Sapper T. N. O’Connor A. . (2022). The effects of a 6-week controlled, hypocaloric ketogenic diet, with and without exogenous ketone salts, on cognitive performance and mood states in overweight and obese adults. Front. Neurosci. 16. doi: 10.3389/fnins.2022.971144. PMID: 36248655 PMC9563373

[B41] Kadowaki T. Yamauchi T. Kubota N. (2008). The physiological and pathophysiological role of adiponectin and adiponectin receptors in the peripheral tissues and CNS. FEBS Lett. 582, 74–80. doi: 10.1016/j.febslet.2007.11.070. PMID: 18054335

[B42] Kang E. Koo J. Jang Y. Yang C. Lee Y. Cosio‐Lima L. M. . (2016). Neuroprotective effects of endurance exercise against high‐fat diet‐induced hippocampal neuroinflammation. J. Neuroendocrinol 28, jne.12385. doi: 10.1111/jne.12385. PMID: 26991447

[B43] Kawai T. Autieri M. V. Scalia R. (2021). Adipose tissue inflammation and metabolic dysfunction in obesity. Am. J. Physiol. Cell Physiol. 320, C375–C391. doi: 10.1152/ajpcell.00379.2020. PMID: 33356944 PMC8294624

[B44] Khalafi M. Hossein Sakhaei M. Kheradmand S. Symonds M. E. Rosenkranz S. K. (2023). The impact of exercise and dietary interventions on circulating leptin and adiponectin in individuals who are overweight and those with obesity: A systematic review and meta-analysis. Adv. Nutr. 14, 128–146. doi: 10.1016/j.advnut.2022.10.001. PMID: 36811585 PMC10103003

[B45] Khoramipour K. Bejeshk M. A. Rajizadeh M. A. Najafipour H. Dehghan P. Farahmand F. (2023). High-intensity interval training ameliorates molecular changes in the hippocampus of male rats with the diabetic brain: the role of adiponectin. Mol. Neurobiol. 60, 3486–3495. doi: 10.1007/s12035-023-03285-z. PMID: 36877358

[B46] Kim T. W. Choi H. H. Chung Y. R. (2016). Treadmill exercise alleviates impairment of cognitive function by enhancing hippocampal neuroplasticity in the high-fat diet-induced obese mice. J. Exerc Rehabil 12, 156–162. doi: 10.12965/jer.1632644.322. PMID: 27419109 PMC4934958

[B47] Kim C. Pinto A. M. Bordoli C. Buckner L. P. Kaplan P. C. Del Arenal I. M. . (2020). Energy restriction enhances adult hippocampal neurogenesis-associated memory after four weeks in an adult human population with central obesity; a randomized controlled trial. Nutrients 12, 638. doi: 10.3390/nu12030638. PMID: 32121111 PMC7146388

[B48] Klein C. Jonas W. Iggena D. Empl L. Rivalan M. Wiedmer P. . (2016). Exercise prevents high-fat diet-induced impairment of flexible memory expression in the water maze and modulates adult hippocampal neurogenesis in mice. Neurobiol. Learn. Mem. 131, 26–35. doi: 10.1016/j.nlm.2016.03.002. PMID: 26968656

[B49] Kolnes K. J. Petersen M. H. Lien-Iversen T. Højlund K. Jensen J. (2021). Effect of exercise training on fat loss—energetic perspectives and the role of improved adipose tissue function and body fat distribution. Front. Physiol. 12. doi: 10.3389/fphys.2021.737709. PMID: 34630157 PMC8497689

[B50] Kullmann S. Goj T. Veit R. Fritsche L. Wagner L. Schneeweiss P. . (2022). Exercise restores brain insulin sensitivity in sedentary adults who are overweight and obese. JCI Insight 7, e161498. doi: 10.1172/jci.insight.161498. PMID: 36134657 PMC9675563

[B51] Lam T. K. T. Schwartz G. J. Rossetti L. (2005). Hypothalamic sensing of fatty acids. Nat. Neurosci. 8, 579–584. doi: 10.1038/nn1456. PMID: 15856066

[B52] Layé S. Nadjar A. Joffre C. Bazinet R. P. (2018). Anti-inflammatory effects of omega-3 fatty acids in the brain: Physiological mechanisms and relevance to pharmacology. Pharmacol. Rev. 70, 12–38. doi: 10.1124/pr.117.014092. PMID: 29217656

[B53] Leckie R. L. Lehman D. E. Gianaros P. J. Erickson K. I. Sereika S. M. Kuan D. C. H. . (2020). The effects of omega-3 fatty acids on neuropsychological functioning and brain morphology in mid-life adults: a randomized clinical trial. Psychol. Med. 50, 2425–2434. doi: 10.1017/S0033291719002617. PMID: 31581959 PMC8109262

[B54] Lee J. An H. S. Shin H. J. Jang H. M. Im C. O. Jeong Y. . (2024). Intermittent fasting reduces neuroinflammation and cognitive impairment in high-fat diet-fed mice by downregulating lipocalin-2 and galectin-3. Nutrients 16, 159. doi: 10.3390/nu16010159. PMID: 38201988 PMC10780385

[B55] Lee C. H. Kim H. J. Lee Y. S. Kang G. M. Lim H. S. Lee S. . (2018). Hypothalamic macrophage inducible nitric oxide synthase mediates obesity-associated hypothalamic inflammation. Cell Rep. 25, 934–946.e5. doi: 10.1016/j.celrep.2018.09.070. PMID: 30355499 PMC6284237

[B56] Lee E. B. Warmann G. Dhir R. Ahima R. S. (2011). Metabolic dysfunction associated with adiponectin deficiency enhances kainic acid-induced seizure severity. J. Neurosci. 31, 14361–14366. doi: 10.1523/JNEUROSCI.3171-11.2011. PMID: 21976521 PMC3195357

[B57] Li Z. Wu X. Gao H. Xiang T. Zhou J. Zou Z. . (2023). Intermittent energy restriction changes the regional homogeneity of the obese human brain. Front. Neurosci. 17. doi: 10.3389/fnins.2023.1201169. PMID: 37600013 PMC10434787

[B58] Liu X. Tang Y. Luo Y. Gao Y. He L. (2024). Role and mechanism of specialized pro-resolving mediators in obesity-associated insulin resistance. Lipids Health Dis. 23, 234. doi: 10.1186/s12944-024-02207-9. PMID: 39080624 PMC11290132

[B59] Liu J. Zaidi A. Pike C. J. (2024). Microglia/macrophage-specific deletion of TLR-4 protects against neural effects of diet-induced obesity. Neuroscience. 22, 214. doi: 10.1186/s12974-025-03534-z. PMID: 41013506 PMC12465214

[B60] Ma J. Liu Y. Hu J. Liu X. Xia Y. Xia W. . (2024). Tirzepatide administration improves cognitive impairment in HFD mice by regulating the SIRT3-NLRP3 axis. Endocrine 87, 486–497. doi: 10.1007/s12020-024-04013-w. PMID: 39222203

[B61] Marcinnò A. Gallo E. Roveta F. Boschi S. Grassini A. Rainero I. . (2022). Decreased resistin plasmatic concentrations in patients with Alzheimer’s disease: A case-control study. Heliyon 8, e11738. doi: 10.1016/j.heliyon.2022.e11738. PMID: 36439765 PMC9694389

[B62] Martin C. K. Carmichael O. T. Carnell S. Considine R. V. Kareken D. A. Dydak U. . (2025). Tirzepatide on ingestive behavior in adults with overweight or obesity: a randomized 6-week phase 1 trial. Nat. Med. 31, 3141–3150. doi: 10.1038/s41591-025-03774-9. PMID: 40555748 PMC12443625

[B63] Mellott E. Faulkner J. L. (2023). Mechanisms of leptin-induced endothelial dysfunction. Curr. Opin. Nephrol Hypertens. 32, 118–123. doi: 10.1097/MNH.0000000000000867. PMID: 36598435 PMC9870925

[B64] Mendes N. F. Velloso L. A. (2022). Perivascular macrophages in high-fat diet-induced hypothalamic inflammation. J. Neuroinflammation 19, 136. doi: 10.1186/s12974-022-02519-6. PMID: 35681242 PMC9185933

[B65] Minato-Inokawa S. Hayashida Y. Honda M. Tsuboi-Kaji A. Takeuchi M. Kitaoka K. . (2023). Association between serum leptin concentrations and homeostasis model assessment-insulin resistance of 2.5 and higher in normal weight Japanese women. Sci. Rep. 13, 8217. doi: 10.1038/s41598-023-35490-0. PMID: 37217782 PMC10203141

[B66] Mishra S. Su Y. Singh S. Kumar A. Hsu F. Deep G. . (2026). Adipose tissue‐derived small extracellular vesicles and blood–brain barrier function in adults with overweight and obesity. Exp. Physiol. 1–12. doi: 10.1113/EP092878. PMID: 41604331 PMC13140706

[B67] Morris A. W. J. Carare R. O. Schreiber S. Hawkes C. A. (2014). The cerebrovascular basement membrane: Role in the clearance of Î^2^-amyloid and cerebral amyloid angiopathy. Front. Aging Neurosci. 6. doi: 10.3389/fnagi.2014.00251. PMID: 25285078 PMC4168721

[B68] Nigro E. Scudiero O. Monaco M. L. Palmieri A. Mazzarella G. Costagliola C. . (2014). New insight into adiponectin role in obesity and obesity-related diseases. BioMed. Res. Int. 2014, 1–14. doi: 10.1155/2014/658913. PMID: 25110685 PMC4109424

[B69] Niraula A. Fasnacht R. D. Ness K. M. Frey J. M. Cuschieri S. A. Dorfman M. D. . (2023). Prostaglandin PGE2 receptor EP4 regulates microglial phagocytosis and increases susceptibility to diet-induced obesity. Diabetes 72, 233–244. doi: 10.2337/db21-1072. PMID: 36318114 PMC10090268

[B70] Obradovic M. Sudar-Milovanovic E. Soskic S. Essack M. Arya S. Stewart A. J. . (2021). Leptin and obesity: Role and clinical implication. Front. Endocrinol. 12. doi: 10.3389/fendo.2021.585887. PMID: 34084149 PMC8167040

[B71] Olivier E. Soury E. Ruminy P. Husson A. Parmentier F. Daveau M. . (2000). Fetuin-B, a second member of the fetuin family in mammals. Biochem. J. 350, 589–597. doi: 10.1042/bj3500589. PMID: 10947975 PMC1221288

[B72] Pan W. Kastin A. J. (2007). Adipokines and the blood-brain barrier. Peptides 28, 1317–1330. doi: 10.1016/j.peptides.2007.04.023. PMID: 17540480 PMC2040301

[B73] Plantera L. Bernhart S. H. Immig K. Leyh J. Ceglarek U. Bechmann I. (2025). Diet-driven microglial activation: Region-specific neuroinflammation in the mouse brain. Brain Sci. 16, 29. doi: 10.3390/brainsci16010029. PMID: 41594751 PMC12839311

[B74] Prévost M. Crépin D. Rifai S. A. Poizat G. Gonçalves M. Van Barneveld F. . (2025). The resistin/TLR4/miR-155-5p axis: a novel signaling pathway in the onset of hypothalamic neuroinflammation. J. Neuroinflamm. 22, 198. doi: 10.1186/s12974-025-03522-3. PMID: 40759954 PMC12323067

[B75] Qi Y. Takahashi N. Hileman S. M. Patel H. R. Berg A. H. Pajvani U. B. . (2004). Adiponectin acts in the brain to decrease body weight. Nat. Med. 10, 524–529. doi: 10.1038/nm1029. PMID: 15077108

[B76] Rajput M. Malik I. A. Methi A. Cortés Silva J. A. Fey D. Wirths O. . (2025). Cognitive decline and neuroinflammation in a mouse model of obesity: An accelerating role of ageing. Brain Behav. Immun. 125, 226–239. doi: 10.1016/j.bbi.2024.12.154. PMID: 39730092

[B77] Ramírez-Carreto R. J. Rodríguez-Cortés Y. M. Torres-Guerrero H. Chavarría A. (2023). Possible implications of obesity-primed microglia that could contribute to stroke-associated damage. Cell. Mol. Neurobiol. 43, 2473–2490. doi: 10.1007/s10571-023-01329-5. PMID: 36935429 PMC10025068

[B78] Rhea E. M. Salameh T. S. Logsdon A. F. Hanson A. J. Erickson M. A. Banks W. A. (2017). Blood-brain barriers in obesity. AAPS J. 19, 921–930. doi: 10.1208/s12248-017-0079-3. PMID: 28397097 PMC5972029

[B79] Rizzo M. R. Fasano R. Paolisso G. (2020). Adiponectin and cognitive decline. Int. J. Mol. Sci. 21, 2010. doi: 10.3390/ijms21062010. PMID: 32188008 PMC7139651

[B80] Rodríguez M. Pintado C. Moltó E. Gallardo N. Fernández-Martos C. M. López V. . (2018). Central s-resistin deficiency ameliorates hypothalamic inflammation and increases whole body insulin sensitivity. Sci. Rep. 8, 3921. doi: 10.1038/s41598-018-22255-3. PMID: 29500410 PMC5834531

[B81] Rohm T. V. Meier D. T. Olefsky J. M. Donath M. Y. (2022). Inflammation in obesity, diabetes, and related disorders. Immunity 55, 31–55. doi: 10.1016/j.immuni.2021.12.013. PMID: 35021057 PMC8773457

[B82] Roy A. Dawson V. L. Dawson T. M. (2025). From metabolism to mind: The expanding role of the GLP-1 receptor in neurotherapeutics. Neurotherapeutics 22, e00712. doi: 10.1016/j.neurot.2025.e00712. PMID: 40738791 PMC12491786

[B83] Ruiz M. Devkota R. Bergh P. Nik A. M. Blid Sköldheden S. Mondejar‐Duran J. . (2024). Aging AdipoR2-deficient mice are hyperactive with enlarged brains excessively rich in saturated fatty acids. FASEB J. 38, e23815. doi: 10.1096/fj.202400293RR. PMID: 38989587

[B84] Rustenhoven J. Jansson D. Smyth L. C. Dragunow M. (2017). Brain pericytes as mediators of neuroinflammation. Trends Pharmacol. Sci. 38, 291–304. doi: 10.1016/j.tips.2016.12.001. PMID: 28017362

[B85] Salas-Venegas V. Flores-Torres R. P. Rodríguez-Cortés Y. M. Rodríguez-Retana D. Ramírez-Carreto R. J. Concepción-Carrillo L. E. . (2022). The obese brain: Mechanisms of systemic and local inflammation, and interventions to reverse the cognitive deficit. Front. Integr. Neurosci. 16. doi: 10.3389/fnint.2022.798995. PMID: 35422689 PMC9002268

[B86] Salem E. A. Alqahtani S. M. El-Shoura E. A. M. Zaghlool S. S. Abdelzaher L. A. Mohamed S. A. M. . (2025). Neuroprotective effects of semaglutide and metformin against rotenone-induced neurobehavioral changes in male diabetic rats. Naunyn Schmiedebergs Arch. Pharmacol. 398, 11919–11931. doi: 10.1007/s00210-025-03920-7. PMID: 40088335 PMC12449337

[B87] Schmitt L. O. Gaspar J. M. (2023). Obesity-induced brain neuroinflammatory and mitochondrial changes. Metabolites 13, 86. doi: 10.3390/metabo13010086. PMID: 36677011 PMC9865135

[B88] Sewaybricker L. E. Huang A. Chandrasekaran S. Melhorn S. J. Schur E. A. (2023). The significance of hypothalamic inflammation and gliosis for the pathogenesis of obesity in humans. Endocr Rev. 44, 281–296. doi: 10.1210/endrev/bnac023. PMID: 36251886 PMC10216879

[B89] Singer K. Maley N. Mergian T. DelProposto J. Cho K. W. Zamarron B. F. . (2015). Differences in hematopoietic stem cells contribute to sexually dimorphic inflammatory responses to high fat diet-induced obesity. J. Biol. Chem. 290, 13250–13262. doi: 10.1074/jbc.M114.634568. PMID: 25869128 PMC4505578

[B90] Stephens T. W. Basinski M. Bristow P. K. Bue-Valleskey J. M. Burgett S. G. Craft L. . (1995). The role of neuropeptide Y in the antiobesity action of the obese gene product. Nature 377, 530–532. doi: 10.1038/377530a0. PMID: 7566151

[B91] Steppan C. M. Bailey S. T. Bhat S. Brown E. J. Banerjee R. R. Wright C. M. . (2001). The hormone resistin links obesity to diabetes. Nature 409, 307–312. doi: 10.1038/35053000. PMID: 11201732

[B92] St-Pierre J. Tremblay M. L. (2012). Modulation of leptin resistance by protein tyrosine phosphatases. Cell Metab. 15, 292–297. doi: 10.1016/j.cmet.2012.02.004. PMID: 22405067

[B93] Stranahan A. M. Guo D. H. Yamamoto M. Hernandez C. M. Khodadadi H. Baban B. . (2023). Sex differences in adipose tissue distribution determine susceptibility to neuroinflammation in mice with dietary obesity. Diabetes 72, 245–260. doi: 10.2337/db22-0192. PMID: 36367881 PMC9871229

[B94] Sun Y. Lv Q. K. Liu J. Y. Wang F. Liu C. F. (2025). New perspectives on the glymphatic system and the relationship between glymphatic system and neurodegenerative diseases. Neurobiol. Dis. 205, 106791. doi: 10.1016/j.nbd.2025.106791. PMID: 39778750

[B95] Takechi R. Lam V. Brook E. Giles C. Fimognari N. Mooranian A. . (2017). Blood-brain barrier dysfunction precedes cognitive decline and neurodegeneration in diabetic insulin resistant mouse model: An implication for causal link. Front. Aging Neurosci. 9. doi: 10.3389/fnagi.2017.00399. PMID: 29249964 PMC5717019

[B96] Thaler J. P. Yi C. X. Schur E. A. Guyenet S. J. Hwang B. H. Dietrich M. O. . (2012). Obesity is associated with hypothalamic injury in rodents and humans. J. Clin. Invest 122, 153–162. doi: 10.1172/JCI59660. PMID: 22201683 PMC3248304

[B97] Tian S. Hong H. Luo X. Zeng Q. Huang P. Zhang M. (2024). Association between body mass index and glymphatic function using diffusion tensor image-along the perivascular space (DTI-ALPS) in patients with Parkinson’s disease. Quant Imaging Med. Surg. 14, 2296–2308. doi: 10.21037/qims-23-1032. PMID: 38545038 PMC10963810

[B98] Till A. Fries C. Fenske W. K. (2022). Brain-to-BAT - and back?: Crosstalk between the central nervous system and thermogenic adipose tissue in development and therapy of obesity. Brain Sci. 12, 1646. doi: 10.3390/brainsci12121646. PMID: 36552107 PMC9775239

[B99] Tomasi D. Wang G. J. Wang R. Backus W. Geliebter A. Telang F. . (2009). Association of body mass and brain activation during gastric distention: Implications for obesity. PloS One 4, e6847. doi: 10.1371/journal.pone.0006847. PMID: 19718256 PMC2729391

[B100] Tomassoni D. Martinelli I. Moruzzi M. Micioni Di Bonaventura M. V. Cifani C. Amenta F. . (2020). Obesity and age-related changes in the brain of the Zucker Lepr fa/fa rats. Nutrients 12, 1356. doi: 10.3390/nu12051356. PMID: 32397542 PMC7284640

[B101] Tremblay E. J. Tchernof A. Pelletier M. Joanisse D. R. Mauriège P. (2024). Plasma adiponectin/leptin ratio associates with subcutaneous abdominal and omental adipose tissue characteristics in women. BMC Endocr Disord. 24, 39. doi: 10.1186/s12902-024-01567-8. PMID: 38481206 PMC10938796

[B102] Valdearcos M. Douglass J. D. Robblee M. M. Dorfman M. D. Stifler D. R. Bennett M. L. . (2017). Microglial inflammatory signaling orchestrates the hypothalamic immune response to dietary excess and mediates obesity susceptibility. Cell Metab. 26, 185–197.e3. doi: 10.1016/j.cmet.2017.05.015. PMID: 28683286 PMC5569901

[B103] Valdearcos M. Robblee M. M. Benjamin D. I. Nomura D. K. Xu A. W. Koliwad S. K. (2014). Microglia dictate the impact of saturated fat consumption on hypothalamic inflammation and neuronal function. Cell Rep. 9, 2124–2138. doi: 10.1016/j.celrep.2014.11.018. PMID: 25497089 PMC4617309

[B104] Walsh J. J. Caldwell H. G. Neudorf H. Ainslie P. N. Little J. P. (2021). Short‐term ketone monoester supplementation improves cerebral blood flow and cognition in obesity: A randomized cross‐over trial. J. Physiol. 599, 4763–4778. doi: 10.1113/JP281988. PMID: 34605026

[B105] Wang J. Li L. Zhang Z. Zhang X. Zhu Y. Zhang C. . (2022). Extracellular vesicles mediate the communication of adipose tissue with brain and promote cognitive impairment associated with insulin resistance. Cell Metab. 34, 1264–1279.e8. doi: 10.1016/j.cmet.2022.08.004. PMID: 36070680

[B106] Wang D. Wu M. Zhang X. Li L. Lin M. Shi X. . (2022). Hepatokine fetuin B expression is regulated by leptin-STAT3 signalling and associated with leptin in obesity. Sci. Rep. 12, 12869. doi: 10.1038/s41598-022-17000-w. PMID: 35896788 PMC9329397

[B107] Wardlaw J. M. Smith C. Dichgans M. (2013). Mechanisms of sporadic cerebral small vessel disease: Insights from neuroimaging. Lancet Neurol. 12, 483–497. doi: 10.1016/S1474-4422(13)70060-7. PMID: 23602162 PMC3836247

[B108] World Health Organization (2025). Obesity and overweight fact sheet (Geneva: World Health Organization (WHO). Available online at: https://www.who.int/news-room/fact-sheets/detail/obesity-and-overweight (Accessed January 5, 2026).

[B109] World Obesity Federation (2025). World obesity atlas 2025 (London: World Obesity Federation). Available online at: https://data.worldobesity.org/publications/?cat=23.

[B110] Xiong S. Wang Q. Chen Y. Du H. Zhao Y. (2024). Leptin limits hepatic lipid accumulation and inflammation via vagal activation of the JAK2-STAT3/AMPK pathway. J. Nutr. Biochem. 134, 109748. doi: 10.1016/j.jnutbio.2024.109748. PMID: 39186956

[B111] Yang L. Chan M. Sheng J. Qi S. Chan B. Shantaram D. . (2025). Decoding adipose–brain crosstalk: Distinct lipid cargo in human adipose‐derived extracellular vesicles modulates amyloid aggregation in Alzheimer’s disease. Alzheimers Dement 21, e70603. doi: 10.1002/alz.70603. PMID: 41036709 PMC12489747

[B112] Yen J. T. Landsell T. A. Laimon-Thomson E. Yen M. Jackson W. F. Dorrance A. M. (2025). High-fat feeding has sex-dependent effects on the structure and biomechanical properties of cerebral parenchymal arterioles and cognitive function. Am. J. Physiol. Heart Circ. Physiol. 329, H1–H15. doi: 10.1152/ajpheart.00295.2024. PMID: 40214009 PMC12199659

[B113] Yigit A. A. Kilinc S. Olcuoglu R. Arnous E. A. (2024). The effects of orlistat on oxidative stress, recognition memory, spatial memory and hippocampal tissue in experimentally induced obesity in rats. Behav. Brain Res. 462, 114894. doi: 10.1016/j.bbr.2024.114894. PMID: 38311071

[B114] Yuan Y. Peng W. Lei J. Zhao Y. Zhao B. Li Y. . (2024). AQP4 endocytosis-lysosome degradation mediated by MMP-9/β-DG involved in diabetes cognitive impairment. Mol. Neurobiol. 61, 8438–8453. doi: 10.1007/s12035-024-04085-9. PMID: 38512439

[B115] Zhan M. Liu X. Xia X. Yang Y. Xie Y. Zhang L. . (2024). Promotion of neuroinflammation by the glymphatic system: A new insight into ethanol extracts from Alisma orientale in alleviating obesity-associated cognitive impairment. Phytomedicine 122, 155147. doi: 10.1016/j.phymed.2023.155147. PMID: 37864890

[B116] Zhang A. Liu Q. Xiong Y. Li J. Xu Y. Song H. . (2025). Tirzepatide reduces body weight by increasing fat utilization via the central nervous system‐adipose tissue axis in male mice. Diabetes Obes. Metab. 27, 2844–2856. doi: 10.1111/dom.16294. PMID: 40000395

[B117] Zhang C. Song J. Zhang W. Huang R. Li Y. J. Zhang Z. . (2025). JAK2/STAT3 signaling in myeloid cells contributes to obesity-induced inflammation and insulin resistance. Cells 14, 1194. doi: 10.3390/cells14151194. PMID: 40801626 PMC12346878

[B118] Zhang W. Xiao D. Mao Q. Xia H. (2023). Role of neuroinflammation in neurodegeneration development. Signal. Transduct Tgt Ther. 8, 267. doi: 10.1038/s41392-023-01486-5. PMID: 37433768 PMC10336149

[B119] Zhao Y. Hong N. Liu X. Wu B. Tang S. Yang J. . (2014). A novel mutation in leptin gene is associated with severe obesity in Chinese individuals. BioMed. Res. Int. 2014, 1–3. doi: 10.1155/2014/912052. PMID: 24707501 PMC3953508

[B120] Zhou Y. Chen Y. Xu C. Zhang H. Lin C. (2020). TLR4 targeting as a promising therapeutic strategy for Alzheimer disease treatment. Front. Neurosci. 14. doi: 10.3389/fnins.2020.602508. PMID: 33390886 PMC7775514

[B121] Zhu Y. He Y. Yang H. Gao Y. Wang Y. Liu P. . (2025). Semaglutide ameliorates diabetes-associated cognitive dysfunction in mouse model of type 2 diabetes. PloS One 20, e0326897. doi: 10.1371/journal.pone.0326897. PMID: 40608828 PMC12225814

[B122] Zhu P. Luo Y. Li Y. Tang J. Liu L. Deng Y. . (2025). Downregulation of AdipoR1 in the hippocampus impairs synaptic function and structure and causes depression-like behavior. Transl. Psychiatry 15, 277. doi: 10.1038/s41398-025-03495-0. PMID: 40796732 PMC12344148

